# Monosubstituted *N*‐Arylhydroxylamine Chemistry: Integrating Contemporary Synthetic Approaches for the Efficient Construction of Diverse Heterocyclic Scaffolds

**DOI:** 10.1002/chem.70973

**Published:** 2026-04-09

**Authors:** Michael G. Kallitsakis, Kyriakos D. Nikopoulos, Ioannis N. Lykakis

**Affiliations:** ^1^ Department of Chemistry Aristotle University of Thessaloniki Thessaloniki Greece

**Keywords:** catalysis, heterocycles, *N*‐arylhydroxylamines, reduction, synthesis

## Abstract

Hydroxylamines represent a structurally diverse class of compounds encompassing both *N‐* and/or *O*‐substituted derivatives, each exhibiting distinct chemical reactivity and synthetic utility. Notably, *N*‐arylhydroxylamines define a unique branch within the hydroxylamine family serving as key intermediates in redox transformations and as reactive platforms for diverse synthetic elaborations. Guided by the distinctive reactivity and unique features of this class, we propose a novel perspective that unifies synthetic approaches with application‐driven insights, emphasizing the strategic significance of monosubstituted *N*‐arylhydroxylamines as versatile building blocks for the construction of complex heterocyclic architectures and a range of valuable organic compounds. By merging recent advances in synthetic strategies with their practical applications, this review aims to provide a comprehensive account of the versatile role of this branch centered on *N*‐arylhydroxylamine motifs in modern organic synthesis.

## Graphical abstract

Monosubstituted N‐arylhydroxylamines represent a unique subclass of hydroxylamines that act as pivotal intermediates in redox transformations and as versatile platforms for further synthetic transformations. They serve as key building blocks in the synthesis of architecturally complex heterocycles and other valuable organic compounds. This review summarizes comprehensive advances in the synthesis and application of monosubstituted *N*‐arylhydroxylamines, highlighting their strategic role in modern organic synthesis and their practical exploitation in heterocycle construction.

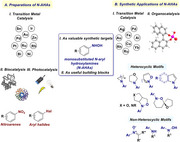

## 1 Introduction

Hydroxylamine and its derivatives represent a pivotal class of nitrogen‐containing compounds serving as versatile precursors for biologically active molecules and a wide range of organic frameworks. Nowadays, they also find diverse applications across pharmaceuticals, agrochemicals, and polymer synthesis, acting as key intermediates in drug and agrochemical production and as stabilizers in polymers. Their unique reactivity profile makes them crucial building blocks in modern organic synthesis. Owing to the characteristic weakness of the N‐O *σ* bond, hydroxylamines readily undergo N‐O bond cleavage, enabling their conversion into a variety of functional groups. Moreover, the hydroxylamine moiety possesses both nitrogen and oxygen nucleophilic centers making it a versatile precursor for the incorporation of N‐O motifs into heterocycles through intra‐ or intermolecular nucleophilic substitution and addition reactions. For this purpose, they have recently attracted significant attention as a platform for developing efficient methodologies for both their synthesis and heterocycle construction.

**FIGURE 1:**
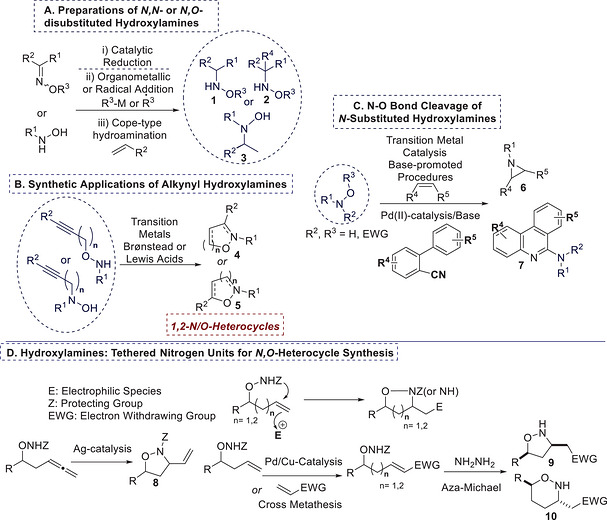
Previous reviews on the synthetic & practical landscape of *N*‐substituted hydroxylamine derivatives (A‐D) [7, 8, 9].

To date, within the field of synthetic organic chemistry, only sporadic resources as review articles and monographs/books, have focused on the hydroxylamine functionality as a point of interest, either systematically outlining the principal strategies for their preparation or distinguishing their role as versatile precursors for the construction of *N*‐heterocycles [1, 2]. In particular, in Patai's comprehensive volume *The Chemistry of Hydroxylamines, Oximes and Hydroxamic Acids*, edited by Z. Rappoport and J. Liebman, a detailed account of *N*‐ and *O*‐substituted hydroxylamines is presented, encompassing classical and modern synthetic approaches, structural features, and reactivity patterns. Within the same volume, M. Lukevics's research team contributed a chapter devoted to the heterocyclic chemistry of hydroxylamines and hydroxamic acids, emphasizing their role as ambident *N*/*O* nucleophilic precursors in substitution, addition, and oxidative pathways [3, 4, 5]. Additionally, regarding hydroxylamine chemistry, a recent review by D. Crich and his research group focuses on developments in synthetic methodologies for the preparation of *N,N*‐di‐, *N,O*‐di‐ and *N,N,O*‐trisubstituted hydroxylamine derivatives **1–3**, highlighting advances in catalytic reductions and alkylation strategies (Figure 1A) [6]. A valuable contribution was made by S. J. Gharpure and coworkers who established alkynyl hydroxylamines as key precursors for 1,2‐*N/O* heterocycles **4–5**, privileged scaffolds found in numerous natural products assessing the mechanistic pathways and stereo‐electronic factors influencing product yield and selectivity (Figure 1B) [7]. In 2023, X.‐H. Ouyang, J.‐H. Li and coworkers placed particular emphasis on recent strategies in N‐O bond cleavage involving both *N*‐ and *O*‐substituted oxime and hydroxylamine derivatives, showcasing their versatility as precursors for nitrogen‐containing heterocycles including aziridines and phenanthridines **6–7** (Figure 1C) [8]. Furthermore, R. W. Bates highlights the strategic use of *N*‐protected hydroxylamines as “tethered nitrogen” units for constructing diverse *N,O*‐heterocycles. Distinct mechanistic approaches, involving allene‐iminium ion cyclizations or utilizing intramolecular aza‐Michael additions in combination with cross‐metathesis, provide efficient access to isoxazolidines, tetrahydro‐1,2‐oxazines and more complex heterocycles **8–10**, demonstrating the synthetic power of hydroxylamine‐based cyclization strategies for alkaloid synthesis (Figure 1D) [9].

**FIGURE 2:**
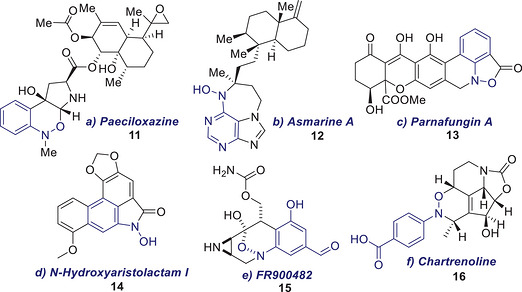
Representative bioactive natural products **11–16** containing masked *N*‐arylhydroxylamine motifs [10, 11, 12, 13, 14, 15, 16, 17, 18].

*N*‐arylhydroxylamines (*N*‐AHAs) key representatives of the hydroxylamine class have recently attracted growing interest due to their broad chemical versatility and functional significance. In recent years, these compounds have found widespread applications across diverse fields, including organic synthesis, heterocyclic chemistry, biological systems, pharmaceutical chemistry, drug discovery and materials science. Importantly, in the context of natural products, *N*‐arylhydroxylamine functionality is predominantly embedded within 1,2‐oxazine hydroxyindole, or isoxazolidine/isoxazolidinone motifs, which can be regarded as “masked” *N*‐arylhydroxylamine architectures reflecting their presence in biologically active molecules [10, 11]. This structural feature is observed across a diverse range of bioactive alkaloids and related natural products. Notably, paeciloxazine **11**, isolated from Paecilomyces BAUA3058, exhibits significant insecticidal and nematocidal activity (Figure 2a) [12, 13]. Similarly, asmarine A **12**, display selective cytotoxicity, where the presence of the *N*‐OH functionality has been demonstrated to be essential for biological activity (Figure 2b) [14]. Parnafungins **13**, represent isoxazolidinone‐containing natural products that act as inhibitors of fungal polyadenylate polymerase, the enzyme responsible for the extension of the poly(A) tail of mRNA transcripts (Figure 2c) [15]. *N*‐Hydroxyaristolactam I (AL‐I‐NOH) **14**, is a critical reactive metabolite formed during the nitro reduction of the nephrotoxic and carcinogenic plant compound aristolochic acid I (AAI) [16]. This metabolite plays a central role in the genotoxic mechanism of AAI, serving as a precursor to DNA‐binding species that generate the characteristic aristolactam (AL)‐DNA adducts (Figure 2d). Meanwhile, FR900482 **15**, isolated from Streptomyces sandaensis, shows potent antitumor activity against multiple cancer cell lines, including mitomycin C‐ and vincristine‐resistant P388 leukemia cells (Figure 2e) [17]. Finally, chartrenoline **16**, a novel alkaloid obtained from Streptomyces chartreusis NA02069, further expands the structural diversity of natural products derived from *N*‐arylhydroxylamine motifs (Figure 2f) [18].

**FIGURE 3:**
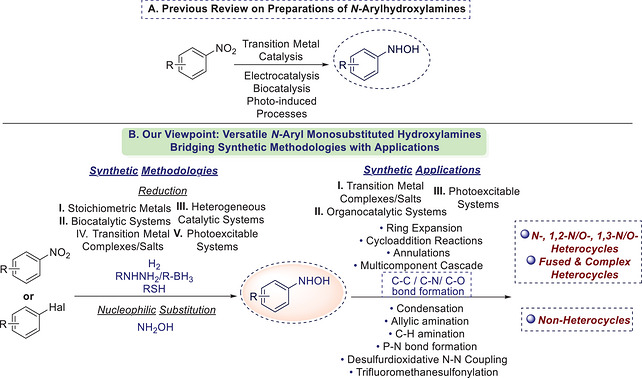
A perspective on monosubstituted *N*‐arylhydroxylamine chemistry: Bridging synthetic strategies with advancing applications [19].

Pertaining to their synthetic exploration, although previous research has focused predominantly on *N*‐ and/or *O*‐(di)‐substituted hydroxylamines, covering either synthetic methodologies or applications in heterocycle synthesis, only a single review has been reported in the literature so far, concentrated on the *N*‐arylhydroxylamine scaffold. From this perspective, L. Wang, Y.‐N. Liu, and coworkers outlined major advances in their preparation via catalytic reduction of aromatic nitro compounds providing a critical assessment of catalytic systems in combination with diverse reductants and hydrogen sources (Figure 3A) [19]. Considering these accounts, our review centrally addresses the connection between the primary synthetic methodologies of monosubstituted *N*‐arylhydroxylamines and their strategic application as versatile precursors to architecturally complex heterocycles and a diverse class of organic compounds. By bridging established and emerging methodologies with their synthetic exploitation, this work provides a valuable overview giving prominence to this *N*‐arylhydroxylamine branch in heterocycle construction and positions them alongside *N*,*N‐* and *N,O‐*disubstituted hydroxylamines as versatile building blocks in organic synthesis (Figure 3B).

With emphasis on the distinct class of monosubstituted *N*‐arylhydroxylamines, this review organizes *N*‐arylhydroxylamine (*N*‐AHA) chemistry into two main categories (chapters **2–3**) based on a systematic analysis of the literature: one addressing their synthetic preparation (**2**) and the other highlighting their synthetic applications (**3**). Specifically, the category (**2**) is further divided into six principal subcategories: **2.1)** nucleophilic substitution reaction, **2.2)** direct catalytic hydrogenation methodologies utilizing molecular hydrogen, **2.3)** stoichiometric metal‐mediated reductions, **2.4)** transfer hydrogenation processes, encompassing catalytic reductions featuring heterogeneous catalysts or transition metal complexes with hydrogen‐donor molecules such as borohydrides, boranes, or hydrazine hydrate, **2.5)** enzyme‐based biocatalysts and **2.6)** noncatalytic photochemical strategies, relying on photoexcited hydrogen‐donor systems under UV‐Vis or visible‐light irradiation via electron transfer (ET), proton‐coupled electron transfer (PCET), or hydrogen atom transfer (HAT) pathways.

In the next section (3), we categorize the distinct applications for accessing diverse libraries of heterocyclic and nonheterocyclic compounds, highlighting *N*‐arylhydroxylamines as key synthetic building blocks. With regard to heterocycle construction, section (3.1) is organized according to the target heterocyclic core, including: **3.1.1)** *β*‐lactams, **3.1.2)** (iso)‐oxazolidines, **3.1.3)** oxazines, **3.1.4)** pyrroles, **3.1.5)** indoles and **3.1.6)** other heterocycles, promoted by transition metal complexes or salts, organocatalytic systems and under basic conditions. Moreover, a detailed analysis of the principal reaction classes is emphasized, which includes: **i)** cycloaddition reactions, such as [3+2] dipolar cycloadditions of in situ‐generated nitrones and nitroso Diels‐Alder processes, providing access to isoxazolidines, oxazolidines, pyrroles, and 1,2‐oxazines, **ii)** ring‐expansion strategies, based on [3+1] cycloadditions, enabling efficient synthesis of *β*‐lactams and their derivatives, **iii)** annulation protocols, exploiting hydroxylamine‐derived nitroso or nitrone intermediates to produce indoles and fused heteroaromatic systems and **iv)** multicomponent cascades, wherein hydroxylamines engage in domino condensations and sequential cycloadditions to construct fused indoles, oxazines and other structurally diverse heterocyclic compounds. Beyond heterocycle construction, *N*‐arylhydroxylamines enable divergent reaction pathways. Accordingly, section (3.2) of this review places special emphasis on versatile methodologies including: **3.2.1)** nitrone preparation, **3.2.2)** allylic amination and C‐H amination of dienes, **3.2.3)** P‐N bond formation exemplified by the synthesis of phosphinic amides and *α*‐hydroxylamino phosphonates, **3.2.4)** direct amide formation via umpolung amide synthesis, **3.2.5)** desulfurdioxidative N‐N coupling and **3.2.6)** *ortho*‐trifluoromethanesulfonylation. Building on the focus on synthetic applications, a dedicated analysis of mechanistic insights will provide a deeper perspective on the underlying pathways that govern *N*‐AHA transformations and guiding the design of more efficient and selective methodologies. Overall, these strategies highlight the central role of *N*‐AHAs as versatile scaffolds in modern synthetic organic chemistry particularly for the development of diverse heterocyclic motifs and several organic molecules of considerable biological interest.

## 2 Synthetic approaches to monosubstituted *N*‐arylhydroxylamine derivatives

**FIGURE 4:**
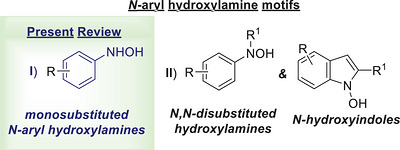
Classification of *N*‐arylhydroxylamines into monosubstituted (**I**) and *N,N*‐disubstituted derivatives(**II**).

To date, two principal classes of *N*‐arylhydroxylamines have been recognized in the literature as valuable synthetic targets: monosubstituted *N*‐arylhydroxylamines (Figure 4 (**I**)) and *N,N*‐disubstituted hydroxylamines (**II**), the latter incorporating *N*‐hydroxyindole‐type derivatives, as well (Figure 4 (**II**)).

Focusing on the scope of this review, we specifically cover a detailed account of synthetic methodologies based on nucleophilic substitution or reduction processes that provide direct and distinguishing access to monosubstituted *N*‐aryl hydroxylamine scaffolds (**I**).

**SCHEME 1:**
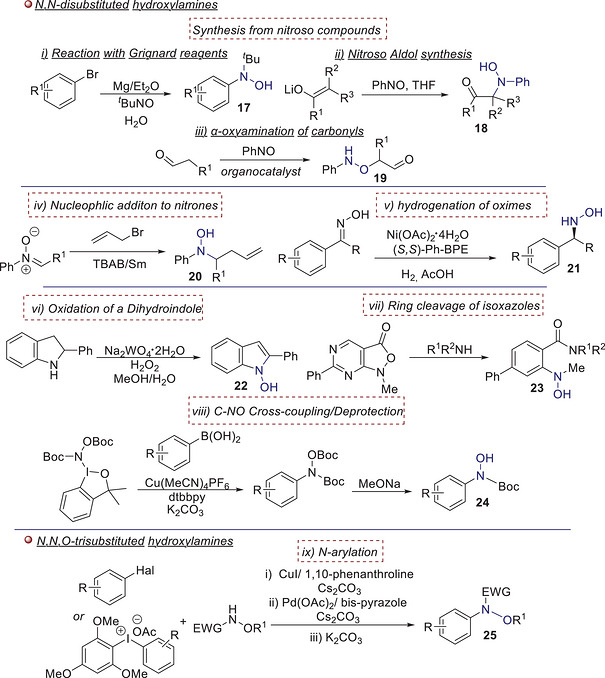
Representative synthetic strategies for *N,N‐di‐ or N,N,O‐*trisubstituted hydroxylamines **17–25** within *N*‐arylhydroxylamine series (**II**) [20, 21, 22, 23, 24, 25, 26, 27].

As a complementary perspective, to offer a synoptic overview of the diversified synthetic methodologies leading to *N,N*‐disubstituted hydroxylamines **II**, **17–24**, herein we disclose in the following scheme the major approaches developed toward this diverse class of *N*‐arylhydroxylamine compounds relying on: i) reactions of nitroso compounds with Grignard or organolithium reagents, ii) the nitroso‐aldol synthesis for example via addition of lithium or tin enolates to nitrosobenzenes, iii) organocatalytic α‐oxyamination of carbonyls, iv), nucleophilic addition to nitrones, v) nickel‐catalyzed asymmetric hydrogenation of oximes, vi) oxidation of dihydroindoles to *N*‐hydroxyindoles **22** under tungstate‐based catalytic protocols, vii) ring cleavage of isoxazoles in the presence of amines and viii) copper‐mediated electrophilic hydroxylamine C‐NO cross‐coupling/deprotection sequence (Scheme 1) [20, 21, 22, 23, 24]. Direct *N*‐arylation strategies (ix) constitute an efficient approach to *N*,*N,O*‐trisubstituted hydroxylamines **25**, achieved under basic conditions using diaryliodonium(III) salts or via palladium‐ and copper‐catalyzed cross‐coupling reactions with aryl halides, thereby enabling the conversion of a broad range of *N*‐ and *O*‐protected hydroxylamines into the corresponding *N*‐arylhydroxylamine candidates (Scheme 1) [25, 26, 27].

### 2.1 Nucleophilic Substitution Reaction

**SCHEME 2:**
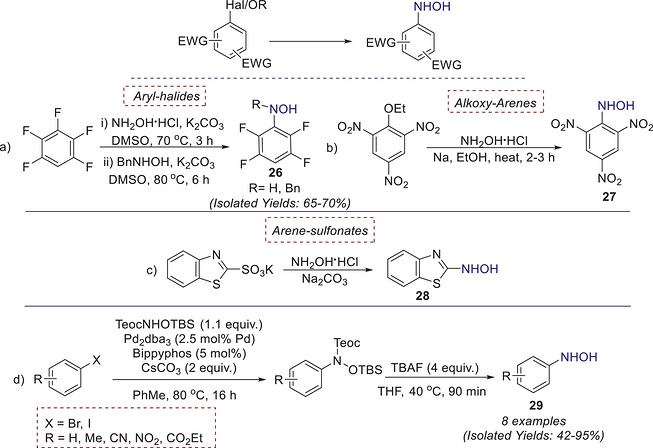
Nucleophilic substitution strategies for access to monosubstituted *N*‐arylhydroxylamine derivatives **26–28** (a–c). Pd‐catalyzed cross‐coupling and deprotection strategy for access to *N*‐arylhydroxylamines **29** (d) [20, 28].

In substitution reactions, hydroxylamines predominantly act as *N*‐nucleophiles, reflecting the higher nucleophilicity of the nitrogen center. In general, two distinct substitution processes can be distinguished for the synthesis of *N*‐arylhydroxylamines: displacement of i) aryl halides and ii) alkoxy groups on electron‐deficient arenes. With respect to aryl halides, nucleophilic substitution with hydroxylamine is feasible on aromatic systems bearing activating electron‐withdrawing groups, as exemplified by the selective reaction of (poly)fluorobenzene with hydroxylamine, affording the corresponding hydroxylamine product **26** under basic conditions (Scheme 2a). In addition to halides, activated alkoxy groups on electron‐poor substrates can serve as suitable leaving groups for displacement by hydroxylamine. Thus, the substitution reaction of ethoxy‐(poly)nitrobenzene derivative with hydroxylamine furnishes the corresponding hydroxylamine **27** (Scheme 2b). As a third variation, the displacement of sulfonate functionality, which acts as an excellent leaving group in nucleophilic substitution can be utilized as a synthetic strategy for the preparation of *N*‐arylhydroxylamine **28** (Scheme 2c) [20].

More recently, D. J. Wuest and coworkers developed a modified two‐step protocol comprising Pd‐catalyzed coupling of aryl halides followed by a deprotection sequence. This methodology relies on the formation of key hydroxylamine intermediates protected with both *tert*‐butyldimethylsilyl (TBS) and [2‐(trimethylsilyl)ethoxy]carbonyl (Teoc) groups (TeocNHOTBS). Importantly, the combination of the Bippyphos ligand with the Pd_2_(dba)_3_ (dibenzylideneacetone) complex proved to be the most effective catalytic system for this transformation. These doubly protected intermediates were efficiently deprotected using TBAF providing access to a broad range of *N*‐arylhydroxylamines **30** under mild conditions that minimize degradation of the final products. Aryl bromides generally afforded higher yields than the corresponding iodides, whereas substrates bearing strongly electron‐donating substituents were not investigated due to the known susceptibility of such *N*‐arylhydroxylamines to degradation (Scheme 2d) [28].

**SCHEME 3:**
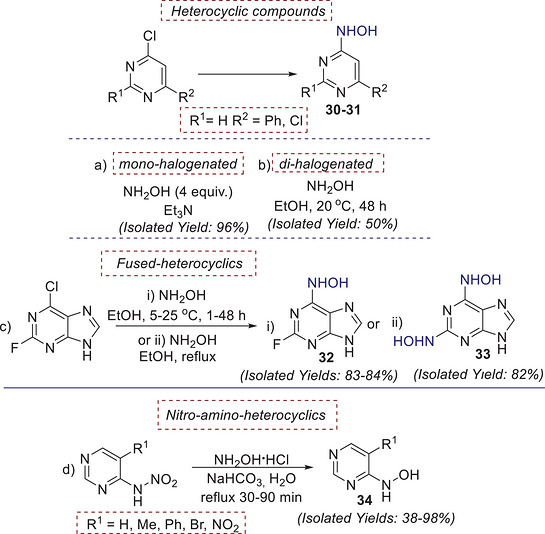
Nucleophilic substitution of heteroaryl substrates with hydroxylamine (a–d) [20].

As a distinct class of compounds, heterocyclic scaffolds represent an important subcategory which follows characteristic reactivity patterns. In this context, the reactions of chloropyrimidines with hydroxylamine are highly dependent on the reaction conditions. As a result, a combination of a fourfold excess of hydroxylamine with triethylamine as base proved optimal for the formation of (hydroxyamino)pyrimidine derivatives **30** (Scheme 3a). This enhanced reactivity has been attributed to the possible formation of intermediate quaternary ammonium salts, which are known to undergo smooth nucleophilic substitutions. On the other hand, dihalogenated heterocyclic analogues, such as pyrimidine or pyridazine substrates, react selectively with hydroxylamine, resulting in substitution of only one halogen atom to afford the corresponding mono(hydroxyamino) derivatives **31** under varying reaction temperature and time (Scheme 3b). Turning our focus to fused heterocyclic systems, nucleophilic aromatic substitution in halogenated purines is preferentially directed to the C‐6 position, a selectivity arising from the greater electrophilic activation of this site. Consequently, a 6‐halo‐substituent on purine can be readily replaced by a hydroxyamino group, as exemplified by the reaction of 2,6‐dihalogenated‐purine precursor with hydroxylamine, which affords the corresponding 6‐(hydroxyamino)‐ purine derivative **32** in high yields at low or ambient temperature. In contrast, under reflux conditions, substitution of both halogen atoms occurs leading to the 2,6‐dihydroxylaminopurine product **33** (Scheme 3c). Furthermore, it has been reported that nitroamino‐pyrimidine derivatives can undergo nucleophilic aromatic substitution under basic conditions, in which the nitro group is displaced upon treatment with hydroxylamine hydrochloride in aqueous solution providing access to hydroxylamine products **34** (Scheme 3d) [20].

### 2.2 Direct Hydrogenation of Nitroarenes

It is well‐established that the reduction of nitro compounds typically proceeds via a direct pathway involving nitroso and hydroxylamine intermediates, leading to the aniline product and making the selective isolation of hydroxylamine derivatives challenging. Moreover, to achieve high yields of *N*‐arylhydroxylamines it is essential to suppress the in situ condensation of nitrosoarenes with *N*‐arylhydroxylamines during nitro reduction process thereby avoiding the subsequent formation of the corresponding dimers. In this context, the formation and stability of reaction intermediates/products generally depend on the kinetics of each step, the concentration of the hydrogen source and the activity of the catalyst. Moreover, one of the critical challenges in this organic transformation lies in achieving the selective reduction of the nitro group in the presence of other reducible functionalities within nitro precursors, while maintaining a high reaction rate and minimizing the formation of by‐products [29, 30, 31, 32, 33, 34].

Traditionally in both industrial and academic settings, the direct hydrogenation of nitroarenes entails high pressures of molecular hydrogen or/and increased temperatures to achieve high conversion typically employing transition‐metal‐based catalytic systems. It is worth noting that the dissociation of H_2_ on the metal surface is both thermodynamically and kinetically favorable. Considering this, the use of support materials such as oxides or carbon‐based matrices can enhance both the selectivity and stability of transition‐metal catalysts by strengthening metal‐support interactions. Moreover, tuning the electronic properties of catalysts can significantly influence the adsorption mode of *N*‐AHAs, thereby suppressing their further hydrogenation and enhancing selectivity toward *N*‐AHAs. Another notable feature of selective hydroxylamine formation is the partial deactivation of the catalyst which prevents further reduction after the absorption of two equivalents of hydrogen allowing hydroxylamine to be released as the final product [29, 30, 31, 32, 33, 34].

#### 2.2.1 Direct Hydrogenation of Nitroarenes with Heterogeneous Catalytic Systems

**SCHEME 4:**
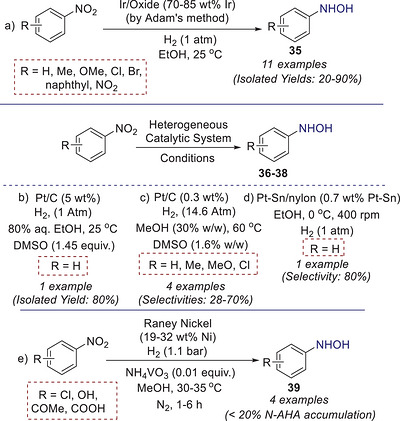
Ir‐oxide and platinum‐based heterogeneous catalytic hydrogenation of nitroarenes to *N*‐hydroxylamines **35–38** (a–d) [35, 36, 37, 38, 39]. Vanadium‐modified Raney nickel for selective formation of *N*‐arylhydroxylamines **39** (e) [40].

In 1966, K. Taya highlighted the efficiency of an iridium oxide‐based catalytic system, prepared via Adams’ method, for the hydrogenation of various aromatic nitro compounds to hydroxylamines **35** under mild conditions, with the reaction monitored until 2 mol of H_2_ was absorbed. In contrast, platinum or palladium catalysts prepared by the same method predominantly yielded aniline, indicating that their faster hydrogenation rates promote complete reduction, thereby suppressing *N*‐AHA formation. While the iridium‐platinum catalyst, prepared via a modification of Adams’ method (70 wt% Ir) promoted the hydrogenation of nitrobenzene faster than the monometallic iridium system, it afforded lower yields of *N*‐AHAs. Several mono‐substituted nitroarenes were efficiently reduced, whereas dinitro compounds underwent regioselective reduction of only one nitro group. K. Taya identified the relatively slow hydrogenation rate of the hydroxylamine intermediate as the critical factor controlling selectivity (Scheme 4a) [35]. Rylander's pioneering investigations on platinum‐catalyzed hydrogenation of nitroaromatic compounds established key principles for the selective partial reduction to *N*‐hydroxylamines **36**, emphasizing the influence of variables such as metal type, metal loading, catalyst promoters, and support on overall yield. Notably, it was reported that platinum‐on‐carbon exhibited significantly higher selectivity toward phenylhydroxylamine compared to unsupported platinum. The rate of hydrogenation increased linearly with catalyst loading within a limited range (50–150 mg), reflecting the importance of hydrogen availability at the catalyst surface. Lower metal loadings favored hydroxylamine formation, while carbon supports offered the optimal combination of reaction rate and selectivity. Among catalyst promoters, the addition of dimethyl sulfoxide (DMSO) markedly enhanced phenylhydroxylamine yield. This effect can be rationalized in terms of competitive adsorption, where phenylhydroxylamine and DMSO interact with catalyst active sites, influencing hydrogenation selectivity and product distribution (Scheme 4b) [36, 37]. Based on Rylander's investigations, A. Rajadhyaksha and coworker further explored the Pt/C system for this selective transformation, systematically evaluating key reaction parameters. Notable features of this process include: i) Addition of a low concentration of DMSO significantly enhanced selectivity by facilitating hydroxylamine desorption and suppressing over‐hydrogenation to aniline, ii) selectivity increased at lower temperatures, iii) selectivity was largely independent of hydrogen pressure, iv) solvent effects correlated with dielectric constants, with higher values favoring PHA selectivity. This is likely due to enhanced solubility of PHA, which promotes its desorption from the catalyst surface, v) for substituted nitroarenes, electron‐releasing groups reduced selectivity, likely due to stronger adsorption of intermediates on the metal surface, increasing the propensity of over‐reduction, vi) other heterogeneous systems, such as Pd/C, showed negligible selectivity for *N*‐hydroxylamines, vii) catalyst loading could be minimized to 0.15 wt% without affecting selectivity, demonstrating the efficiency of this heterogeneous approach for the controlled synthesis of *N*‐arylhydroxylamines **37** (Scheme 4c) [38]. Furthermore, Z. Poltarzewski and coworkers investigated the selective hydrogenation of nitrobenzene over a Pt‐Sn/nylon catalytic system. Low Sn content promoted catalytic activity through activation of oxygen‐containing groups, whereas higher Sn/Pt ratios led to platinum poisoning, rendering hydrogen activation the rate‐determining step. Although, the selectivity toward phenylhydroxylamine **38** is scarcely affected by the Sn/Pt ratio. The reaction followed pseudo‐zero‐order kinetics with respect to nitrobenzene, with specific rate constants largely unaffected by catalyst loading. Temperature strongly influenced PHA selectivity, reflecting differences in adsorption enthalpies between the nitro substrate and *N*‐AHA. Spectroscopic analysis confirmed the formation of metallic platinum, with the authors suggesting that tin addition decreased platinum particle size, thereby increasing metal surface area and enhancing catalytic activity at low Sn concentrations (Scheme 4d) [39]. In 2000, M. Studer and coworkers evaluated the role of vanadium modifiers in the selective hydrogenation of electron‐deficient aromatic nitro compounds over Raney nickel, focusing on the control of *N*‐arylhydroxylamine accumulation, reaction rate, catalyst deactivation and dehalogenation. Unmodified Raney nickel exhibited high *N*‐AHA accumulation, whereas vanadium promoters enabled modulation of this accumulation, either increasing or decreasing it depending on the nature of the modifier and the substrate. Supported or unsupported ammonium metavanadate (NH_4_VO_3_) proved particularly effective, providing enhanced reaction rates together with controlled hydroxylamine accumulation under substrate‐specific optimized conditions. The performance of vanadium was directly linked to its oxidation state, with salts in the IV and V states showing the highest efficiency. The results indicated that vanadium modifiers act primarily as disproportionation catalysts: those that reduced *N*‐AHA accumulation generally induced partial catalyst deactivation, likely through poisoning of hydrogenation sites, whereas modifiers that increased the overall hydrogenation rate tended to promote higher CHB accumulation. The sequence of catalyst treatment was also critical; optimal performance was achieved when the catalyst was first activated, then promoted and utilized immediately, whereas incorporation of the promoter during pre‐treatment led to significant loss of hydrogenation activity. Dehalogenation remained largely unaffected by vanadium promotion and became feasible only after complete consumption of both the nitroarene and the corresponding arylhydroxylamine derivatives. Overall, vanadium‐modified Raney nickel efficiently controlled *N*‐AHA accumulation, lowering it to below 20% across the examined nitro substrates, thereby enabling selective formation of the targeted arylhydroxylamine products **39** (Scheme 4e) [40].

**SCHEME 5:**
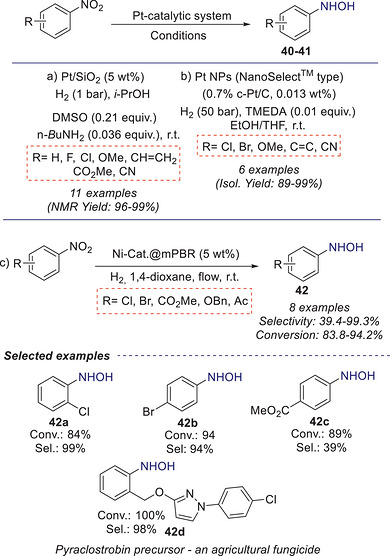
Selective hydrogenation of nitroaromatics using Pt/SiO_2_ with DMSO and primary amines (a), or NanoSelect‐type Pt NPs (b) or a continuous‐flow protocol based on a passivated RANEY nickel catalyst (c) [41, 42, 43].

An efficient heterogeneous catalytic system consisting of 5 wt% platinum on silica was applied by H. Yasuda and coworkers to the selective formation of *N*‐AHAs **40** from the corresponding nitroaromatics. The direct hydrogenation process is achieved in the presence of molecular hydrogen at room temperature in isopropyl alcohol. A key feature is that the synergistic combination of DMSO and an amine significantly enhances the reaction efficiency. The catalytic system is compatible with a wide range of nitroaromatics and demonstrates excellent tolerance toward other reducible functionalities such as alkenes. Notably, substrates bearing electron‐donating substituents (e.g., methoxy groups) required longer reaction times than their electron‐deficient counterparts. The promotive effect of amines was generally observed in the order: tertiary < secondary < primary. This effect is proposed to arise from activation of the nitro group by the amine proton partially assisted by the increased hydride character of hydrogen on platinum. Additionally, diamines were more effective than monoamines. The presence of DMSO is also critical as its strong adsorption on the platinum surface is suggested to suppress further hydrogenation of *N*‐arylhydroxylamines **40**, thereby enhancing selectivity. The catalyst can be recovered by filtration and reused with only a slight decrease in activity (Scheme 5a) [41]. In 2013, D. Vogt and coworkers introduced a heterogeneous catalytic protocol based on NanoSelect‐type platinum nanoparticles, prepared via a reduction‐deposition method and investigated their performance in this chemoselective transformation. The catalysts proved highly effective with selectivity strongly influenced by temperature, hydrogen pressure, solvent and amine additives. Low temperatures favored *N*‐AHA formation while increasing hydrogen pressure further enhanced selectivity. Solvent optimization studies identified an EtOH/THF mixture as optimal, while the addition of TMEDA significantly enhanced the reaction yields by facilitating desorption of *N*‐AHAs **41** from the catalyst surface and stabilizing key intermediates through hydrogen bonding.The Pt‐based system demonstrated broad tolerance to electron‐withdrawing and electron‐donating substituents, along with reducible sensitive moieties (Scheme 5b) [42]. Focusing on the selective hydrogenation of nitroarenes to *N*‐arylhydroxylamines **42**, W.‐K. Su and coworkers outlined a continuous‐flow protocol using a passivated RANEY‐nickel catalyst, with 1,4‐dioxane identified as the optimal solvent at room temperature. The use of a micro‐packed bed reactor enabled highly efficient heterogeneous catalysis with the RANEY‐nickel pretreated with a mixture of aqueous ammonia and DMSO particularly effective at a particle size of 400–500 mesh. The methodology was exemplified through the synthesis of a key hydroxylamine intermediate **42d** of the antifungal agent pyraclostrobin achieving excellent conversion and selectivity. In terms of mechanism, the authors suggested that hydrogen adsorption on the catalyst surface leads to the generation of active hydrogen species responsible for the selective hydrogenation. Catalyst regeneration was accomplished via an in‐column strategy (Scheme 5c) [43].

**SCHEME 6:**
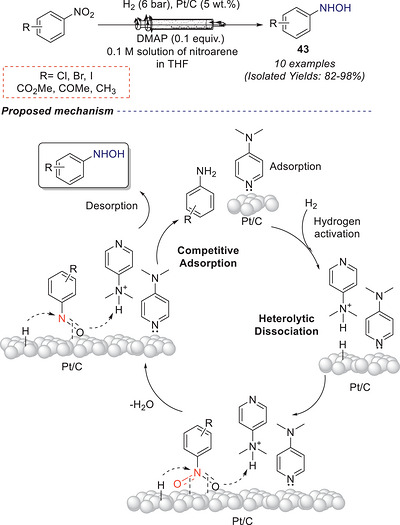
Continuous‐flow selective hydrogenation of nitroarenes to *N*‐arylhydroxylamines **43** using a Pt/C/H_2_ catalytic system in combination with DMAP [44].

Alternatively, F. Liu, J. Chen and coworkers identified DMAP, notable for its high basicity, as a unique additive that enhances both activity and selectivity (up to >99%) in Pt/C‐catalyzed hydrogenation of nitroarenes under mild continuous‐flow conditions. This protocol tolerates a broad substrate scope, including electron‐donating substituents, carbonyls and halogens. In terms of mechanism, DMAP was proposed to exhibit a dual effect: i) the pyridine nitrogen adsorbs strongly on the Pt surface, inducing electron transfer that enriches the positive charge of Pt and modulates its electronic environment and ii) the nitrogen of the dimethylamino group forms a frustrated Lewis pair (FLP) with the Pt active site, enabling heterolytic H_2_ activation. The combined effects of competitive adsorption, electron transfer and FLP formation facilitate selective reduction to *N*‐arylhydroxylamines **43** while suppressing over‐reduction to anilines (Scheme 6) [44].

#### 2.2.2 Direct Hydrogenation of Nitroarenes with Transition Metal Complexes

**SCHEME 7:**
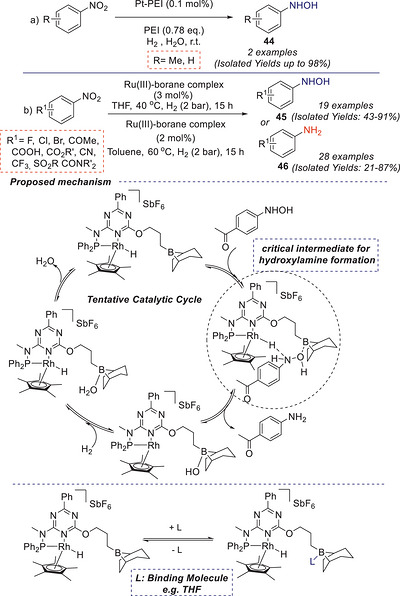
Hydrogenation of aromatic nitro compounds using a platinum(II)‐polyethyleneimine (Pt(II)‐PEI) complex (a) or an adaptive Rh(III)‐borane catalyst (b) with molecular hydrogen [45, 46].

Furthermore, the incorporation of suitable coordinating organic ligands into transition metal complexes can also enhance the efficiency and selectivity of nitroarene hydrogenation under mild reaction conditions. In this context, N. Izakovich and coworkers reported a platinum(II)‐polyethyleneimine (Pt(II)‐PEI) complex, reduced by sodium borohydride as an efficient catalyst for the hydrogenation of aromatic nitro compounds under mild conditions. Using molecular hydrogen at atmospheric pressure and temperatures between 10–70°C, the Pt‐ system selectively converts nitroarenes into the corresponding *N*‐arylhydroxylamines **44**. The addition of excess amount of PEI significantly improves both the rate and selectivity, an effect attributed to its ability to form molecular complexes with nitroarenes and thereby lower the activation barrier for the electron‐transfer step. It has been suggested that PEI can coordinate to *N*‐arylhydroxylamine intermediates inhibiting their further hydrogenation and thus favoring high selectivity toward hydroxylamine products. The catalytic protocol was applied to two nitrobenzene derivatives in aqueous and aqueous‐alcoholic media, affording excellent yields. Notably, the stability of the Pt(II)‐PEI complex is pH‐dependent with decomposition occurring below pH 6.96 (Scheme 7a) [45]. Later, C. Werlé and his research group developed an adaptive Rh(III)‐borane catalyst that utilized molecular hydrogen for the chemoselective hydrogenation of nitroarenes. The system exhibited solvent‐controlled selectivity: in THF the selective formation of the *N*‐arylhydroxylamine derivative **45** was achieved, whereas in toluene the reaction proceeded directly to the aniline product **46**. High yields were obtained across a broad substrate scope with excellent tolerance toward readily reducible functional groups such as ketones, amides, sulfones and alkenes which remained unaffected. The origin of this adaptivity lies in the Lewis acidic borane ligand incorporated in the secondary coordination sphere. In noncoordinating solvents (toluene), the borane ligand activates the hydroxylamine intermediate enabling its further reduction to aniline. In coordinating solvents such as THF, however solvent‐borane adducts are formed through oxygen‐boron interactions. This interaction competes with substrate binding and thereby suppresses N‐O bond cleavage, directing the reduction toward the hydroxylamine derivative. Mechanistic investigations revealed a direct pathway involving: i) formation of a rhodium‐hydride intermediate species, ii) hydride transfer to the boron‐activated substrate and solvent competition with the substrate for borane coordination, which critically determines the reaction outcome. Further mechanistic insights based on density functional theory (DFT) calculations indicated a three‐step pathway into this divergent, selective hydrogenation process: 1) in the absence of the borane ligand hydroxylamine remains unreactive toward nucleophilic attack by the rhodium‐hydride intermediate, 2) the borane ligand then acts as a hydroxide acceptor, orienting the hydroxylamine to facilitate hydride transfer, 3) in a concerted process the hydride is transferred from the rhodium center to the nitrogen atom, facilitating N‐O bond cleavage thereby promoting aniline formation (Scheme 7b) [46].

### 2.3 Stoichiometric Metal‐mediated Reductions

**SCHEME 8:**
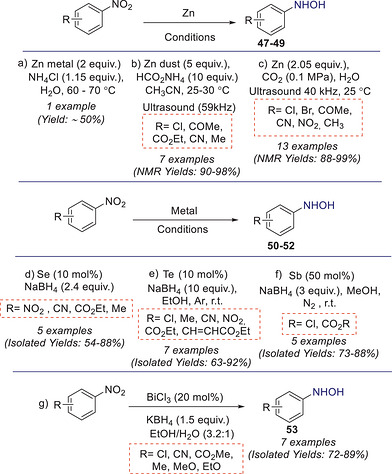
Reduction of nitroarenes to *N*‐arylhydroxylamines **47–49** using Zn/NH_4_Cl, Zn/HCO_2_NH_4_ under ultrasound and Zn in a CO_2_‐H_2_O system (a–c) [48, 49, 50]. Selective Synthesis of *N*‐arylhydroxylamines **50–53** using Se, Te, or Sb metals (d–f) or bismuth trichloride with sodium/potassium borohydride (g) [53, 54, 55, 56].

In 1894, *N*‐phenylhydroxylamine (*N*‐PHA) was independently discovered by Wohler and Bamberger during the zinc‐mediated reduction of nitrobenzene. This work led to the identification of the Bamberger rearrangement, in which N‐PHA undergoes acid‐promoted conversion to 4‐aminophenol. The reaction remains a classic example of an electrophilic rearrangement, providing valuable insight into the introduction of nucleophilic groups onto aromatic compounds [47]. In addition, T. Kamm reported the chemoselective reduction of nitroarenes to *N*‐arylhydroxylamines **47** using a twofold excess of zinc metal in combination with ammonium chloride in an aqueous suspension. Optimal results were obtained when the reaction was conducted at 65–70 °C (Scheme 8a) [48]. Following Kamm's methodology, R. W. Lu and coworkers developed an ultrasound‐assisted approach utilizing zinc dust in combination with an excess of ammonium formate in acetonitrile. Under ultrasonic irradiation at room temperature, *N*‐AHAs **48** were obtained in high yields under mild reaction conditions. The reduction process was strongly solvent‐dependent: in methanol, complete conversion to the corresponding anilines was observed, while in acetonitrile the reaction selectively yielded *N*‐arylhydroxylamine products (Scheme 8b) [49]. Furthermore, J. Jiang and coworkers demonstrated the promoting effect of ultrasound on the transfer hydrogenation of nitroarenes to *N*‐arylhydroxylamines **49** using zinc in an environmentally benign CO_2_‐H_2_O system. Under the optimized conditions comprising a Zn‐to‐nitrobenzene molar ratio of 2.2, with ultrasonic irradiation, at ambient temperature and CO_2_ pressure a variety of substituted *N*‐arylhydroxylamines was obtained in excellent yields (88%–99%) with high selectivity (91%–100%). The application of ultrasound provided several advantages, including enhanced yields, shorter reaction times, and reduced zinc consumption. The reaction takes place through several key steps: 1) Transfer of nitrobenzene from the solution to the Zn^0^ surface, 2) Adsorption of nitrobenzene onto the Zn^0^ surface, 3) Surface‐mediated reduction of nitrobenzene, involving the formation of *N*‐phenylhydroxylamine, 4) Desorption of the products from the metallic surface and 5) Diffusion of the products back into the solution. Remarkably, ultrasonic irradiation facilitates these steps by enhancing mass transfer and increasing the active zinc surface area through particle rupture. Moreover, the acidity of the CO_2_‐H_2_O system appears to play a significant role, as lower temperatures where CO_2_ solubility is greater lead to higher conversion efficiencies (Scheme 8c) [50].

### 2.4 Catalytic Transfer Hydrogenation Processes

#### 2.4.1 Borohydride/Borane‐mediated Transfer Hydrogenation

It is well‐establishes that the NaBH_4_‐mediated catalytic transfer hydrogenation involves several essential mechanistic steps: i) in aqueous or alcoholic media, NaBH_4_ undergoes B‐H bond cleavage to generate molecular hydrogen and borate species (B(OH)_4_^−^), ii) adsorption of molecular hydrogen onto the catalyst surface, where iii) it is activated through interaction with the metal forming surface‐bound hydride species ([M]‐H) responsible for reducing the nitro compounds [29, 33]. In the case of amine boranes (RNH_2_BH_3_), the literature distinguishes between two mechanistic pathways: i) classical transfer hydrogenation processes in which the double hydrogen transfer originates from both the amine and borane moieties and ii) nonclassical transfer hydrogenation processes wherein an initial hydroboration from the amine‐borane complex is followed by solvolysis [51, 52].

##### 2.4.1.1 Borohydride/Borane‐mediated transfer hydrogenation with metals or metal salts

In 1986, S. Uchida and coworkers reported the selective synthesis of *N*‐AHAs **50** using catalytic amounts of selenium metal in combination with sodium borohydride as the reducing agent. Generally, 10 mol% of the selenium catalyst and an excess of sodium borohydride were applied in ethanol. Kinetic studies indicated that electron‐withdrawing substituents on the aromatic ring accelerate the reduction, whereas electron‐donating groups tend to retard it. The catalytically active hydrogen species is proposed to be hydrogen selenide anion, generated in situ through the reaction of selenium metal with sodium borohydride (Scheme 8d) [53]. In the same year, the same research group demonstrated that tellurium could also serve as an effective metal catalyst for the reduction of nitroarenes via transfer hydrogenation in the presence of sodium borohydride. This approach allowed the preparation and isolation of a variety of para‐substituted nitroarenes under inert atmosphere at room temperature. Mechanistic studies suggested that sodium hydrogentelluride, generated in situ under basic conditions could serve as the catalytically active species. The reactivity of sodium hydrogentelluride was strongly medium‐dependent: acidic conditions favored the formation of azoxybenzenes, while basic conditions promoted efficient reduction to the corresponding *N*‐arylhydroxylamines **51** (Scheme 8e) [54]. Alternatively, S. Wu and coworkers reported the selective synthesis of *N*‐arylhydroxylamines with an antimony metal/NaBH_4_ catalytic system. Notably, the nature of the antimony species strongly influenced the reaction outcome: whereas elemental antimony promoted selective formation of *N*‐AHAs **52**, the use of SbCl_3_ led to complete reduction, yielding the corresponding amines (Scheme 8f) [55]. More recently, Zi‐Peng Yao and coworkers achieved the selective reduction of aromatic nitro compounds to the corresponding hydroxylamines **53** by combining potassium borohydride with bismuth trichloride in a water/ethanol mixture at room temperature. The method is carried out under mild conditions and affords *N*‐arylhydroxylamines in high yields (72%–89%), while leaving other reducible functionalities unaffected. The efficiency of this transformation is strongly influenced by the substituents’ electronic nature: electron‐withdrawing groups favor the reduction, while strongly electron‐donating groups induce oxidation of the initially formed *N*‐AHAs, producing the corresponding azoxy derivatives (Scheme 8g) [56].

##### 2.4.1.2 Borohydride/Borane‐mediated reductions with heterogeneous catalytic systems

**SCHEME 9:**
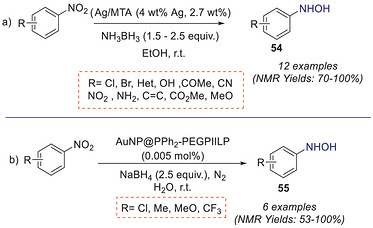
Chemoselective reduction of nitroarenes using µesoporous Ag/MTA composites/NH_3_BH_3_ (a) or phosphine‐decorated, polymer‐immobilized ionic liquid (PIIL)‐stabilized Au NPs with NaBH_4_ (b) [57, 58].

In 2016, I. N. Lykakis and coworkers developed mesoporous 4% Ag/MTA composites and demonstrated their efficiency in the selective reduction of nitroarenes. The Ag/TiO_2_ assemblies exhibited large surface areas and narrow mesopores (7.1–7.4 nm) features that contribute significantly to their high catalytic performance. The heterogeneous catalytic system exhibited divergent activity depending on the borohydride reducing agent. By varying the amount of ammonia borane, *N*‐arylhydroxylamines **54** were obtained in short reaction times with excellent yields (70%–99%). The Ag‐based system displayed broad functional group tolerance, leaving esters, nitriles, alkenes, and lactones intact and it reduced only one nitro group in dinitro substrates, highlighting its regioselectivity. Mechanistic studies revealed that cleavage of the B‐H bond in ammonia borane generates active [Ag]‐H species, which are proposed to mediate the selective reduction of nitroarenes to the corresponding *N*‐arylhydroxylamines. A Hammett‐type kinetic analysis indicated that substrates bearing electron‐withdrawing substituents are reduced more rapidly than those with electron‐donating groups. The positive ρ values are consistent with a transition state involving hydride transfer, in which the developing negative charge is stabilized by electron‐withdrawing substituents (Scheme 9a) [57]. In a more recent study, S. Doherty, J. G. Knight and coworkers reported phosphine‐decorated, polymer‐immobilized ionic liquid (PIIL)‐stabilized gold nanoparticles as an efficient catalytic system for the selective reduction of nitroarenes at low catalyst loadings. Varying the reaction conditions significantly affected the product distribution. Performing the reaction in water at room temperature under a nitrogen atmosphere favored selective formation of *N*‐arylhydroxylamines **55**. Higher temperatures or prolonged reaction times promoted further reduction to anilines, whereas reactions in air produced azoxy compounds through reversible condensation of *N*‐AHAs, demonstrating the system's temperature/oxygen‐dependent selectivity. The introduction of PEG into the polymer network significantly enhanced catalyst performance by increasing hydrophilicity, improving water dispersibility and providing additional stabilization through interactions between ether oxygen atoms and nanoparticle surfaces. This modification enabled exceptionally high efficiency, with TON and TOF values reaching 100,000 and 73,000 h^−1^, respectively. It was further suggested that the PIIL component influenced nanoparticle growth, contributing to the high selectivity achieved (Scheme 9b) [58].

**SCHEME 10:**
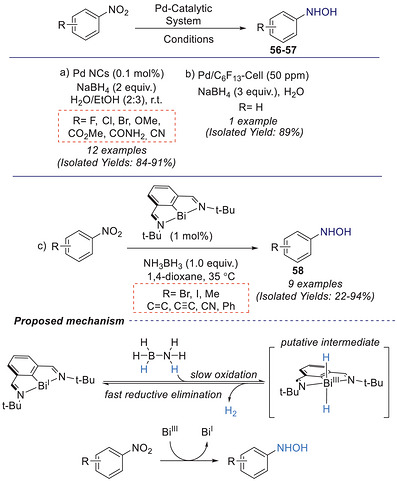
Pd nanoclusters (Pd NCs) and Pd NPs supported on perfluorinated cellulose/NaBH_4_ catalytic systems toward the chemoselective synthesis of *N*‐arylhydroxylamines **56–57** (a–b) [59, 60]. Reduction of nitroarenes catalyzed by a Bi(I) complex with NH_3_BH_3_ (c) [61].

In the following year, M. Ma and coworkers reported a catalytic tandem protocol toward this goal, in which NaBH_4_ served as the reducing agent and palladium nanoclusters (Pd NCs), generated in situ from Pd(OAc)_2_, acted as the catalyst. The ultrasmall Pd NCs (1.3 ± 0.3 nm) were stabilized by surface‐coordinated nitroarenes which suppressed growth and aggregation thereby preserving high catalytic activity. The reaction selectivity pattern proved highly dependent on catalyst loading, solvent nature and the amount of reducing agent. By fine‐tuning these parameters, the methodology could be directed toward five distinct classes of nitrogen‐containing products such as *N*‐AHAs, anilines, azoxy‐, azo‐, and hydrazo‐compounds, with yields up to 98% within 30 min at room temperature. Under optimized conditions (0.1 mol% Pd(OAc)_2_ in H_2_O/EtOH), *N*‐arylhydroxylamine derivatives **56** were isolated in 84%–91% yields within 10 min. The Pd‐catalytic system displayed broad functional group tolerance and could be recovered and reused for at least five cycles without significant loss of activity (Scheme 10a) [59]. In parallel, aiming at the selective synthesis of *N*‐phenylhydroxylamine (PHA, **57**) from nitrobenzene, C. Cai and coworkers successfully prepared palladium nanoparticles (Pd NPs) supported on perfluorinated cellulose via a solid state method. The enhanced catalytic performance was attributed both to the high activity of the Pd NPs and to the influence of fluorine‐containing groups on the support. Characterization studies revealed that perfluorinated cellulose possesses a high surface compared to natural cellulose which facilitates the adsorption and stabilization of ultrasmall Pd NPs. The reaction occurs efficiently in water under mild conditions, requiring only very low catalyst loadings (25–50 ppm), achieving high conversion and selectivity in under 30 min at room temperature. The tunable hydrophilicity/hydrophobicity of the modified cellulose support was found to play a key role: a more hydrophobic surface preferentially adsorbs nitrobenzene over PHA, thereby preventing over‐reduction to aniline. From a mechanistic point of view the authors proposed that the excellent performance arises from a combination of factors: i) the ultrafine size of Pd NPs ensures intimate contact between active sites and substrate, ii) the presence of F‐containing groups enriches electrons on the metal surface, increasing the Pd° content and activity and iii) the perfluoroalkyl‐modified support improves selective adsorption, enhancing both activity and selectivity (Scheme 10b) [60].

##### 2.4.1.3 Borane‐mediated transfer hydrogenation with transition metal complexes

Pnictogen‐based complexes and particularly bismuth(I) complexes exhibit unique catalytic properties due to their low oxidation state and soft Lewis acidity. These complexes can undergo redox cycles between Bi(I) and Bi(III) facilitating electron transfer processes essential for hydrogenation reactions. Their reactivity is often enhanced by the presence of electron‐donating ligands, which stabilize the low‐valent bismuth center. Building on this concept, J. Cornella's research group introduced a well‐defined bismuth(I) complex (1 mol%) as an efficient catalyst to accomplish this chemoselective reaction. Reactions were performed in 1,4‐dioxane at 35 °C using ammonia borane (AB) as the hydrogen donor. The methodology exhibits broad functional group tolerance, including substrates bearing alkynes, alkenes, and nitriles, which were reduced to the corresponding hydroxylamine products **58** in moderate to excellent yields. The mechanistic pathway for the Bi(I)‐catalyzed reduction of nitroarenes involves: 1) activation of ammonia borane (AB): the Bi(I) complex promotes slow dehydrogenation of AB, generating highly reactive Bi(III) hydride intermediates, 2) hydride transfer: the Bi(III) hydride transfers a hydride to the nitroarene substrate, reducing it to the corresponding N‐arylhydroxylamine, 3) catalyst regeneration: the transfer of hydride restores the Bi(I) species, completing the Pn(I)/Pn(III) redox cycle (Scheme 10c) [61].

**SCHEME 11:**
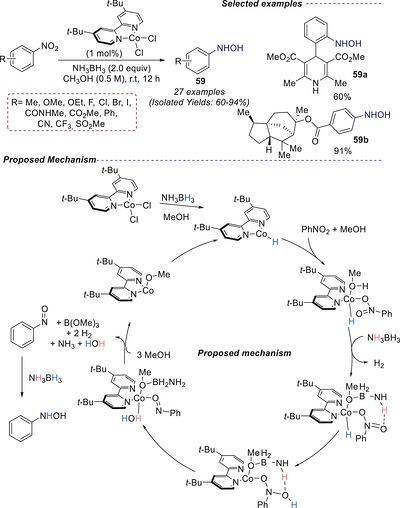
Chemoselective synthesis of *N*‐arylhydroxylamines **59** catalyzed by a dipyridyl‐based Co complex using ammonia‐borane [62].

D. Kong, L. Zhao and coworkers developed a cobalt‐catalyzed transfer hydrogenation in methanol under mild conditions, with ammonia borane serving as the hydrogen donor. The catalytic reaction proceeds with 1 mol% of the dipyridyl‐based Co complex at room temperature exhibiting excellent selectivity toward *N*‐AHAs **59**. This system displays broad functional group tolerance, demonstrating high efficiency toward complex natural products and drug derivatives. Density functional theory (DFT) studies revealed that the N‐H bond of NH_3_BH_3_ plays a crucial role in hydrogen transfer, while methanol participates only indirectly and does not provide hydrogen atoms directly for nitroarene reduction, a distinct pathway from classical solvent‐mediated protonolysis. The catalytic cycle primarily involves: i) reduction of the dtbbpy‐Co‐Cl_2_ by NH_3_BH_3_, ii) a hydride transfer through a five‐membered cyclic transition state formed between the B‐H bond of ammonia borane and the cobalt center, identified as the rate‐determining step; iii) subsequent N‐H hydrogen transfer from the amino group of ammonia borane to the oxygen atom of the reduced nitro group, iv) followed by a noncatalytic reaction of the nitrosoarene intermediate with ammonia‐borane to yield the *N*‐arylhydroxylamine derivative. Remarkably, selectivity arises from the unique square‐pyramidal coordination environment of the cobalt complex with NH_3_BH_3_ and hydroxylamine, which suppresses further N‐O bond reduction (Scheme 11) [62].

#### 2.4.2 Hydrazine‐Mediated Transfer Hydrogenation of Nitroarenes

In general, the proposed reaction pathway for hydrazine‐mediated transfer hydrogenation processes involves three crucial steps: i) coordination of N_2_H_4_∙H_2_O to the catalyst surface, ii) strong metal‐hydride interactions on the catalyst, which lower the N‐H bond cleavage barrier and iii) decomposition of N_2_H_4_∙H_2_O on the metal surface to generate a diimide intermediate (HN═NH) which subsequently produces only N_2_ and H_2_ as benign by‐products [63, 64].

##### 2.4.2.1 Hydrazine‐mediated transfer hydrogenation of nitroarenes with heterogeneous catalytic systems

**SCHEME 12:**
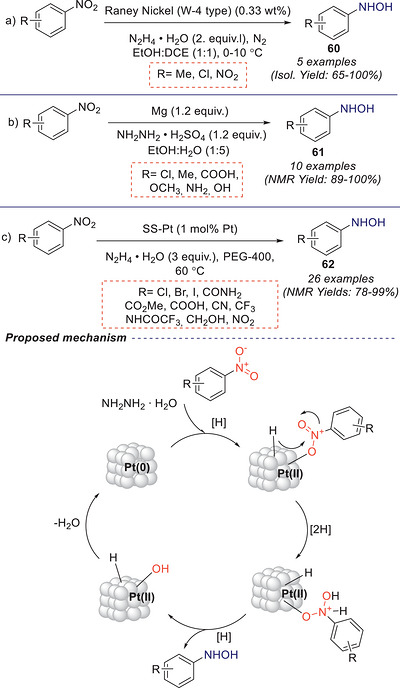
Synthesis of *N*‐arylhydroxylamines **60–61** using Raney Ni/NH_2_NH_2_·H_2_O or magnesium/hydrazine sulfate catalytic systems (a, b) [65, 66]. Hydrazine‐mediated chemoselective synthesis of *N*‐arylhydroxylamines **62** using solid‐supported platinum(0) nanoparticles (SS‐Pt) (c) [67].

**SCHEME 13:**
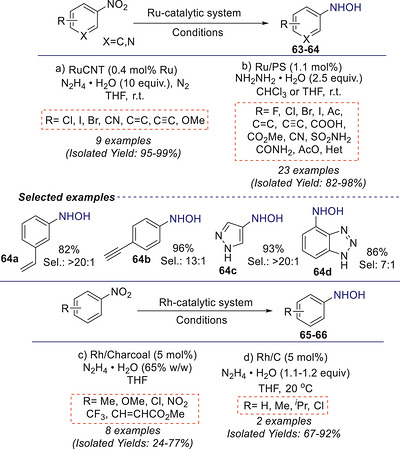
Selective reduction of nitroarenes using ruthenium nanoparticles stabilized on CNTs or polystyrene‐supported ruthenium nanoparticles with NH_2_NH_2_·H_2_O (a,b) [68, 69]. Synthesis of *N*‐arylhydroxylamines **65–66** using a Rh/C/ NH_2_NH_2_·H_2_O catalytic system (c,d) [20, 70].

The first example of hydrazine‐mediated reduction of nitroarenes was reported by N. R. Ayyangar and coworkers in 1984. Using Raney nickel as the catalyst and hydrazine hydrate as the reducing agent the authors successfully isolated five unprotected hydroxylamines **60**, while seventeen distinct products were converted in situ to their benzohydroxamic acid derivatives due to instability under the reaction conditions. It is worth noting that the reaction temperature must be maintained between 0–10 °C and a mixture of ethanol and dichloroethane was used as the ideal solvent medium (Scheme 13a) [65]. More recently, M. A. Pasha and H. M. Nanjundaswamy introduced the first use of hydrazine sulfate as a reducing agent for the efficient synthesis of *N*‐arylhydroxylamines. The reduction was performed in an ethanol/water mixture with magnesium metal turnings, allowing the transformation to be completed within 1–2 min and affording excellent yields of up to 96% (Scheme 13b) [66]. Subsequently, P. Das and coworkers reported the chemoselective reduction of nitroarenes to *N*‐arylhydroxylamines **61** using solid‐supported platinum(0) nanoparticles (SS‐Pt). The catalyst was prepared on Amberlite IRA 900 Cl^−^ resin via a reduction/deposition technique. Hydrazine hydrate served as the hydrogen donor, and PEG‐400 was utilized as a green solvent. The reduction was efficiently carried out under optimal conditions using 1 mol % Pt and an excess of hydrazine hydrate at 60  °C. The system exhibited excellent tolerance toward a wide range of functional groups, while leaving C═C and C≡C bonds intact Moreover, the nanocatalyst efficiently converted both electron‐withdrawing and electron‐donating substituted nitroarenes to the corresponding *N*‐arylhydroxylamines in high yields and it was successfully applied on a 10 g scale. Regarding recyclability, the SS‐Pt catalyst could be reused up to 10 times without significant loss of activity. Mechanistically, the reduction is proposed to proceed via hydride transfer through a coordinated hydroxyammonium intermediate, with hydrogen‐bond‐assisted decomposition of hydrazine on the SS‐Pt surface and coordination of the nitro group to in situ‐generated Pt^2+^ supporting the observed selectivity for *N*‐arylhydroxylamines **62** (Scheme 12) [67].

Carbon nanotubes (CNTs) have been widely applied for providing high accessibility of the active phase, tunable porosity and hydrophobicity, and the ability to fine‐tune metal‐support interactions, enhancing both activity and selectivity. Exploiting these features, E. Doris, I. N. N. Namboothiri and coworkers established a heterogeneous ruthenium catalyst consisting of Ru nanoparticles stabilized on CNTs in the presence of a large excess of hydrazine hydrate. The reaction outcome was strongly solvent‐dependent: in tetrahydrofuran (THF), *N*‐arylhydroxylamines **63** were selectively formed, whereas in water the corresponding anilines were obtained. This effect was attributed to solvent modulation of the substrate's adsorbed state on the catalyst surface, variations in hydrogen and reactant concentrations, and in water a lowered activation energy favoring full reduction. The catalyst was recyclable up to five times without significant loss of activity and showed broad tolerance toward potentially reducible functional groups, including alkenes and alkynes. Generally, this layer‐by‐layer assembled Ru/CNT nanohybrid enabled efficient, selective, and solvent‐controlled reduction of nitroarenes under mild conditions with excellent isolated yields (Scheme 13a) [68]. A few years later, D. J. Michaelis and coworkers developed polystyrene‐supported ruthenium nanoparticles as an efficient catalytic system for the transfer hydrogenation of nitroarenes. The solvent was found to play a crucial role in determining product selectivity, as organic solvents capable of dissolving the polymer‐supported nanocatalyst favored the formation of hydroxylamines **64** over the corresponding anilines. Chloroform was identified as the optimal medium, providing excellent selectivity and high isolated yields with 1.1 mol% catalyst loading and hydrazine hydrate as the hydrogen source. It is noteworthy that the catalytic system exhibited broad functional group tolerance, including toward potentially reducible functionalities such as C═C and C≡C bonds and demonstrated high chemoselectivity even in the presence of heteroaromatic substrates (Scheme 13b) [69]. Alternatively, nitroarenes can be rapidly reduced under relatively mild conditions with hydrazine hydrate in the presence of rhodium catalysts supported on carbon materials, selectively affording *N*‐arylhydroxylamine derivatives **65** at low temperature (20°C). The best results are generally obtained in tetrahydrofuran (THF) or in THF mixed with protic polar solvents such as water or ethanol. Regarding the isolation of the *N*‐arylhydroxylamine, treatment of the crude reaction mixture with cyclohexane facilitates its separation. For example, R. A. W. Johnstone and coworkers reported that the use of a 5 mol% rhodium‐on‐charcoal catalyst in combination with hydrazine hydrate in tetrahydrofuran enables this selective transformation. The method is applicable to the synthesis of *N*‐arylhydroxylamines **66** bearing diverse substitution patterns, including strongly electron‐withdrawing groups such as NO_2_ and CF_3_ as well as acrylate moieties (Scheme 13c, d) [20].

**SCHEME 14:**
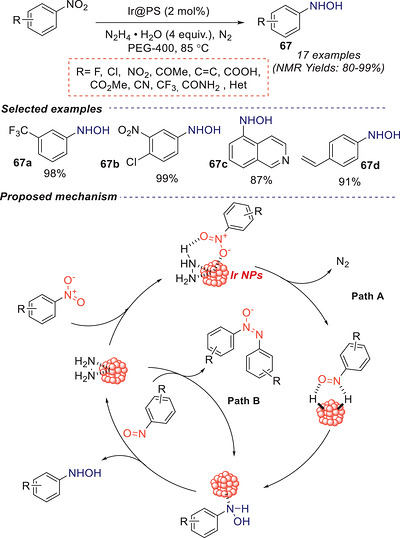
Chemoselective reduction of nitroarenes using a polystyrene‐stabilized iridium nanoparticle (Ir@PS)/hydrazine hydrate catalytic system in PEG‐400 [71].

In 2021, P. Das and coworkers developed a polystyrene‐stabilized iridium nanoparticle catalyst (Ir@PS) to enable this reduction process. Hydrazine hydrate in excess was the only effective hydrogen donor, and PEG‐400 was identified as a suitable green solvent. The catalyst displayed broad substrate scope, efficiently reducing both electron‐rich and electron‐deficient nitroarenes to *N*‐arylhydroxylamines **67**, while tolerating a wide range of reductively sensitive functional groups without by‐product formation. The catalyst demonstrated broad generality, selectively converting even dinitro‐substituted substrates to the corresponding nitro‐monosubstituted *N*‐arylhydroxylamines with excellent regioselectivity. Mechanistic studies suggested that the reduction proceeds via a nitrosobenzene intermediate, with noncovalent interactions between hydrazine and the substrate at the catalyst surface facilitating N‐H bond cleavage and dissociation of hydrazine into H_2_ and N_2_. PEG‐400 further promotes the process by assisting hydrogen adsorption onto the catalytic surface (Scheme 14) [71].

**SCHEME 15:**
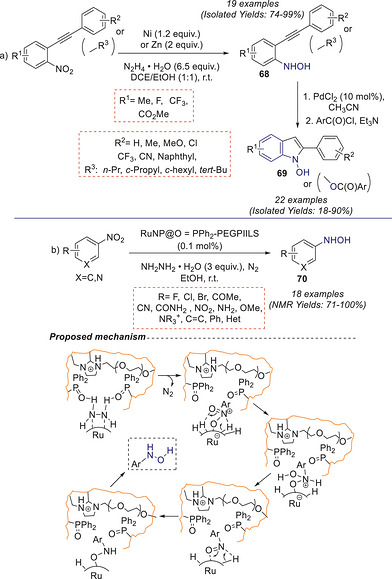
Zn‐ or Ni dust/NH_2_NH_2_·H_2_O reduction of o‐alkynylnitrobenzenes to *N*‐arylhydroxylamines **68** followed by Pd‐catalyzed cyclization to *N*‐hydroxyindoles **69** (a) [72]. Hydrazine‐mediated reduction of nitroarenes using phosphine oxide‐decorated, polymer‐immobilized, ionic liquid‐stabilized Ru NPs (b) [73].

In parallel, S. Shin and coworkers developed a novel Pd‐catalyzed strategy for the synthesis of *N*‐hydroxyindole derivatives **69**, valuable motifs in pharmaceuticals and organic materials. The method initially involves with the reduction of o‐alkynylnitrobenzenes to *N*‐arylhydroxylamines **68**, affording high to excellent yields (74%–99%) with nickel or zinc powder and an excess of hydrazine hydrate as the reducing agent. The reaction proceeds smoothly at room temperature, with a mixture of dichloroethane/ethanol identified as the optimal solvent system. This protocol demonstrates excellent tolerance toward a variety of easily reducible functional groups and is applicable to both electron‐deficient and electron‐rich substrates. Inspired by Cacchi's cyclization of ortho‐alkynyl anilines, the resulting *o*‐alkynyl hydroxylamines **68** undergo PdCl_2_‐catalyzed electrophilic cyclization in acetonitrile to afford C2‐substituted *N*‐hydroxyindoles **69**. To circumvent stability issues, certain *N*‐hydroxyindole derivatives were in situ protected via an *O*‐acylation procedure (Scheme 15a) [72]. More recently, S. Doherty's research group reported a phosphine oxide‐decorated, polymer‐immobilized ionic liquid‐stabilized ruthenium nanoparticle catalyst for the hydrazine‐mediated reduction of nitroarenes to *N*‐AHAs **70** under ambient conditions. The catalyst preparation involved three main steps: 1) synthesis of the polymer support by AIBN‐initiated polymerization of a PEG‐imidazolium monomer, imidazolium cross‐linker and diphenyl(4‐vinylphenyl)phosphine oxide, 2) impregnation of the polymer with RuCl_3_ to form the catalyst precursor and 3) in situ reduction of the precursor with NaBH_4_ to yield the active ruthenium nanoparticle catalyst, RuNP@O = PPh_2_‐PEGPIILS (0.1 mol%). The reaction progress was strongly influenced by solvent and temperature: ethanol was identified as the optimal medium, whereas mixtures of water and ethanol or higher temperatures decreased selectivity favoring complete reduction to aniline. The catalyst was confirmed to be heterogeneous via hot filtration tests and could be reused up to five times without significant loss of activity. Remarkably, the catalytic system displayed broad functional group tolerance and high chemoselectivity, exemplified by the preferential reduction of one nitro group in dinitrobenzene and the chemoselective conversion of 3‐nitrostyrene to *N*‐(3‐vinylphenyl)hydroxylamine under optimal conditions. From a mechanistic perspective, the reduction is proposed to occur via: i) generation of active Ru‐H species from hydrazine, facilitated by hydrogen bonding between the phosphine oxide on the polymer and surface‐bound hydrazine and ii) a direct reduction pathway for the nitroarene (Scheme 15b) [73].

#### 2.4.3 Transfer Hydrogenation with Miscellaneous Reducing Agents

**SCHEME 16:**
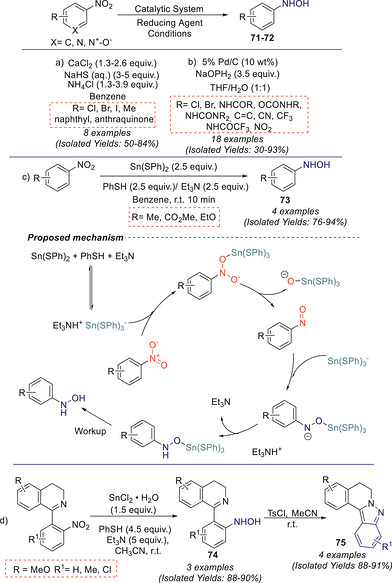
Selective synthesis *N*‐arylhydroxylamines **71–72** using a CaCl_2_/NaHS/NH_4_Cl or Pd/C/NaOPH_2_ catalytic system (a,b) [74, 70]. Tin(II) benzenethiolate/PhSH/Et_3_N or Tin(II)Cl_2_ PhSH/Et_3_N as efficient catalytic systems for the reduction of nitroarenes (c,d) [75, 76].

In 1921, R. D. Haworth and A. Lapworth developed a catalytic method using sodium hydrogen sulfide as a hydrogen donor, in combination with CaCl_2_ and NH_4_Cl in benzene as the solvent, to efficiently reduce several nitroaromatics to the corresponding *N*‐AHAs **71** (Scheme 16a) [74]. Later, R. A. W. Johnstone and coworkers reported the use of sodium phosphinite as an efficient hydrogen source to enable this selective transformation, with palladium on carbon (Pd/C) as a catalyst significantly reducing reaction times and further enhancing the overall efficiency of the reduction process. The method employed a two‐phase solvent system (THF‐H_2_O) to suppress over‐reduction to amines, achieving high selectivity particularly for nitroarenes bearing electron‐withdrawing substituents and affording the corresponding hydroxylamine derivatives **72** in moderate to high yields (Scheme 16b) [70]. Additionally, J. Vilarrasa and coworkers outlined a chemoselective methodology utilizing a tin(II) benzenethiolate‐based complex as efficient catalyst for the transfer hydrogenation of nitroarenes. The cooperative combination of PhSH and Et_3_N proved crucial for the process, promoting the rapid transformation in apolar solvents and affording *N*‐AHAs **73** in good to high yields. Mechanistic studies: 1) indicate that the reaction proceeds via deoxygenation of the nitroarene to the corresponding nitrosoarene, followed by chelation with the tin species to produce the desired product, 2) confirmed the involvement of Sn(SR)_3_^−^ species, which are responsible for the reduction (Scheme 16c) [75]. More recently, B. Kundu developed a mild, efficient, one‐pot protocol for the cyclization of nitro‐aryl substrates via N‐N bond formation, proposing the involvement of a crucial hydroxylamine intermediate. This approach enabled the distinct preparation of *N*‐arylhydroxylamines **74** using tin(II) chloride as the catalyst, in the presence of thiophenol and triethylamine, efficiently producing the desired products within 15 min at room temperature. The basic conditions play a dual role: they hinder complete reduction of the nitro group to the amine while promoting formation of the cyclization product, which is kinetically favored. The cyclization process was achieved via the addition of tosyl chloride, leading to the formation of the corresponding indazole derivatives **75** in high yields (Scheme 16d) [76].

Across both heterogeneous and homogeneous catalytic platforms, selective *N*‐arylhydroxylamine formation via hydrogenation or transfer hydrogenation is governed by precise control over the final reduction step. In heterogeneous systems, selectivity arises from a) surface‐controlled hydrogen availability or b) H‐donor‐generated metal‐hydride species, achieved through i) low metal loadings or ii) partial catalyst deactivation, together with iii) metal‐support interactions, iv) solvent‐dependent surface processes and v) competitive adsorption by additives, which tune the electronic properties of active sites, weaken N‐O bond activation and promote rapid desorption of hydroxylamine intermediates, particularly under mild temperatures and in polar medium systems. Complementarily, transition‐metal complexes rely on: i) redox cycling of the metal center to regenerate active hydride species, ii) leveraging electronically and sterically controlled ligand design to regulate hydride transfer, iii) with secondary coordination sphere effects, including hydrogen bonding or Lewis acid‐base interactions from functional group substituents on ligands and iv) solvent coordination further stabilizing key intermediates and suppressing N‐O bond cleavage. In both approaches, precise control over hydrogen activation and intermediate stabilization is central to preventing over‐reduction and enabling the selective formation of *N*‐AHAs.

### 2.5 Biocatalytic Hydrogenation

**SCHEME 17:**
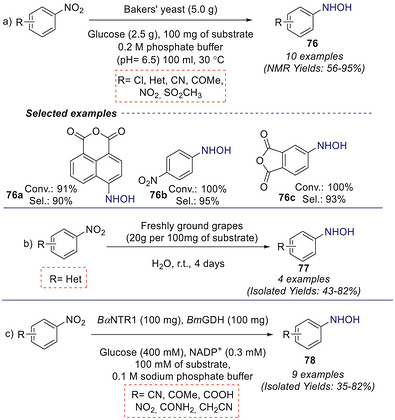
Biocatalytic synthesis of *N*‐arylhydroxylamines **76–78** using *baker's yeast*, grape (*Vitis vinifera L*.) cells or nitroreductase BaNTR1 in the presence of NADP^+^ (a–c) [77, 78, 79].

In 2004, F. Li and coworkers explored baker's yeast (*Saccharomyces cerevisiae*) as an efficient biocatalyst to achieve this chemoselective process. Aromatic nitro compounds bearing electron‐withdrawing groups were chemoselectively reduced to the corresponding hydroxylamines **76** with good to excellent yields under mild conditions. It should be noted that the transformation was strongly dependent on the amount of yeast: higher yeast loadings promoted over‐reduction of the hydroxylamine products to the corresponding anilines, whereas lower yeast amounts enabled highly chemo‐selective formation of the desired hydroxylamines. The methodology demonstrated applicability to a broad range of substrates, including various nitro‐substituted heterocycles and tolerated several easily reducible functional groups (Scheme 17a). More recently, the same research group switched the biocatalyst to grape (*Vitis vinifera L*.) cells to achieve this transformation. Interestingly, the reduction of certain nitro substrates bearing lactam or imide functionalities led to the efficient formation of the corresponding hydroxylamines **77** within four days exhibiting higher chemo‐selectivity compared to the previously reported yeast‐mediated reduction (Scheme 17b) [77, 78]. Nitroreductases (NTRs) are flavin‐containing enzymes that utilize NADH or NADPH as electron donors and can serve as efficient biocatalysts in a variety of organic reduction processes. With respect to this, Jian‐He Xu and coworkers investigated the activity and selectivity of nitroreductases for the synthesis of *N*‐AHAs **78**. Among the enzymes tested, BaNTR1, produced by *Bacillus amyloliquefaciens* exhibited optimal activity at pH 7.0 with NADP^+^ as the electron‐supplying cofactor. Overall, this enzymatic system achieves conversions and chemo‐selectivities of 99%, efficiently reducing substrates bearing electron‐withdrawing groups. Remarkably, the system exhibits high regioselectivity, selectively reducing only one nitro group in dinitro substrates (Scheme 17c) [79].

### 2.6 Photoinduced Transfer Hydrogenation of Nitroarenes

Over the past decade, visible‐light‐driven photocatalysis has emerged as a promising and versatile strategy for promoting efficient, clean and selective redox transformations in modern organic synthesis. Among various substrates, nitroarenes exhibit distinctive photophysical properties that enable their direct excitation under light irradiation, facilitating photoinduced reduction pathways. Upon photoexcitation, nitroarenes can generate a reactive triplet ^3^(π,π*) excited state which effectively engages in hydrogen atom transfer (HAT) processes in the presence of suitable hydrogen donors thus promoting the reduction mechanism. Moreover, promotion of an electron from a nonbonding orbital on oxygen into the π‐system induces a radical‐like character to the oxygen atoms, enhancing the electrophilic nature of the (n,π*)‐excited nitro group and further facilitating the reduction process [80, 81, 82, 83, 84].

**SCHEME 18:**
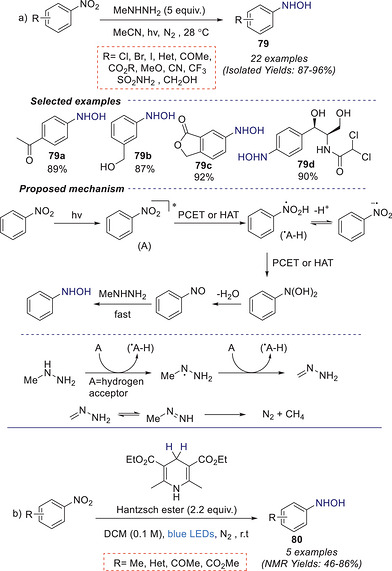
Noncatalytic, methylhydrazine or Hantzsch ester‐mediated (a‐b), photoinduced synthesis of *N*‐arylhydroxylamines **79–80** [85, 86].

To date, I. N. Lykakis, M. G. Kallitsakis and coworkers exploited the photochemical potential of hydrazine derivatives as hydrogen‐donor reagents in this selective transformation. In this regard, they demonstrated a metal‐free, photo‐induced reduction of nitroarenes to *N*‐arylhydroxylamines **79**, mediated by a large excess of methylhydrazine under UV‐Vis light irradiation, maintaining the temperature at 28°C. In the absence of any catalyst, the reaction proceeded smoothly to afford the corresponding *N*‐arylhydroxylamines **79** in excellent yields and selectivity. Among the solvents tested, acetonitrile (MeCN) was identified as the optimal medium, promoting high conversion across a broad range of substrates including heteroaromatic derivatives and the antibiotic compounds azomycin and chloramphenicol. In terms of mechanism, the reaction is suggested to initiate through photoexcitation of the nitroarene, followed by a proton‐coupled electron transfer (PCET) or hydrogen atom transfer (HAT) process between the excited nitroarene and methylhydrazine. The resulting nitrosobenzene intermediate then undergoes a nonphotochemical reduction to produce the corresponding *N*‐arylhydroxylamine. Under the optimized photochemical conditions, a radical degradation pathway of methylhydrazine was also proposed. Overall, this noncatalytic, photo‐induced reduction methodology constitutes a mild, efficient and selective approach for the synthesis of *N*‐AHAs **79**, featuring broad substrate scope, functional group tolerance and short reaction times (Scheme 18a) [85]. As an alternative photochemical approach, A. J. von Wangelin and coworkers disclosed a photochemical strategy using Hantzsch ester as the hydrogen donor to efficiently mediate this reduction process. It is worth noting that the metal‐free process is carried out efficiently under blue LED irradiation in the presence of a Hantzsch ester as the hydrogen source in DCM at ambient temperature over 18 h. The reactions afforded hydroxylamine products in 46%–86% yields with electron‐withdrawing substituents reacting more rapidly than those with electron‐donating substituents. It should be noted the reducing agent is activated via visible‐light irradiation (blue LEDs), generating highly reducing excited‐state species that induce partial hydrogenation through either a single‐electron transfer (SET) or proton‐coupled electron transfer (PCET) mechanism. Mechanistic studies suggested the formation of an electron donor‐acceptor (EDA) complex between the substrate and the Hantzsch ester, supported by an observed bathochromic shift, which facilitates photoinduced electron transfer to produce the hydroxylamine products **80** (Scheme 18b) [86].

**SCHEME 19:**
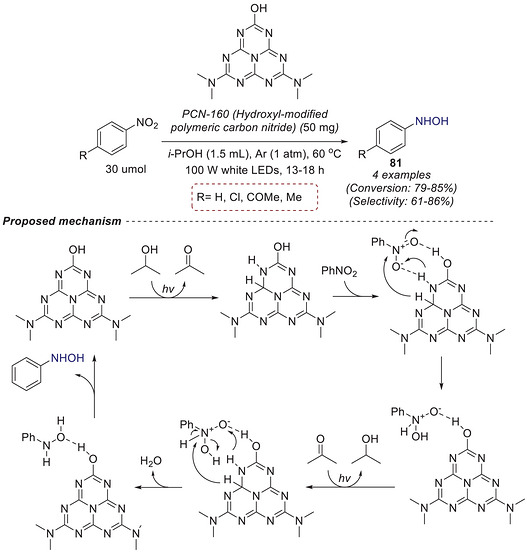
Polycarbonate nitride‐promoted photoinduced reduction of nitroarenes under white LEDs [87].

Polycarbonate nitride (PCN) is a nitrogen‐rich, conjugated polymeric material with efficient visible‐light absorption and tunable surface functionalities, making it a versatile metal‐free photocatalyst. Leveraging these properties, Z. Zheng, J. Jia, X. Ke, X. Gu and coworkers developed PCN catalysts enriched with hydroxyl (‐OH) groups for the selective reduction of nitro compounds to *N*‐arylhydroxylamines **81**. Using isopropyl alcohol/water as a solvent mixture under visible‐light irradiation, the system achieved 80% PHA (phenylhydroxylamine) selectivity. Of particular note, the ‐OH functional groups facilitate nitrobenzene adsorption, suppress electron‐hole recombination and provide protons essential for hydrogen transfer. They also enhance electron's energy that favor hydrogenation of nitroarenes with electron‐withdrawing groups, while electron‐donating groups retarded the reaction (Scheme 19) [87].

**SCHEME 20:**
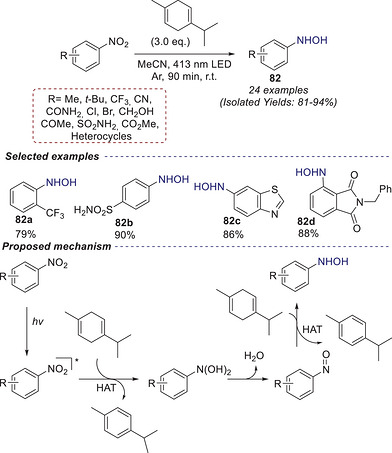
Photochemical *γ*‐terpinene‐mediated reduction of nitroarenes [88].

In a more recent study, X. Huang and C. Cai reported a green and efficient photochemical approach, utilizing plant‐derived γ‐terpinene as a hydrogen donor under 413 nm LED irradiation at room temperature to achieve this selective reduction. This method efficiently converts diverse nitroarenes into *N*‐arylhydroxylamines **82** in high isolated yields of up to 94%, in the absence of metal catalysts or additives. The transformation proceeds with excellent selectivity and exhibits a broad substrate scope, tolerating a wide range of functional groups. Interestingly, several nitro‐substituted heterocycles were well‐tolerated providing access to their corresponding *N*‐arylhydroxylamines in 73%–90% yields. Mechanistic investigations suggest a hydrogen atom transfer (HAT) pathway, wherein γ‐terpinene serves as a hydrogen donor to the nitroarene substrate, generating *p*‐cymene as a by‐product (Scheme 20) [88].

**SCHEME 21:**
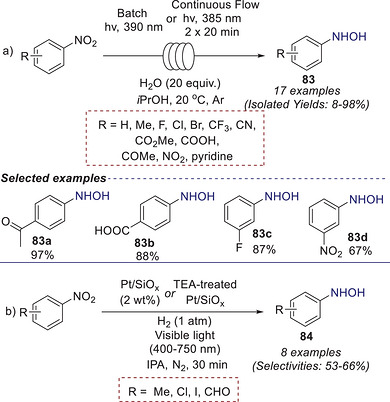
Photoreduction of nitroaromatics by iPrOH in a continuous flow process (a) [89] Visible‐light‐driven synthesis of *N*‐AHAs **84** using nanocrystals (Pt NCs) supported on silica nanospheres (b) [90].

With regard to light‐driven processes, J.‐M. Paris, J. Cossy and coworkers reported the photoreduction of substituted nitro (hetero)arenes using isopropanol (*i*PrOH) under inert atmosphere in both batch and continuous flow processes. The reaction efficiently afforded *N*‐(hetero)arylhydroxylamines **83**, with higher chemoselectivity observed for nitroarenes bearing electron‐withdrawing groups, while other easily reducible functional groups remained unaffected. Optimal results were obtained at 390 nm in batch and at 385 nm in continuous flow after two consecutive 20 min cycles, at 20°C. Mechanistically, nitroarenes abstract hydrogen from *i*PrOH, which is oxidized to acetone; the presence of excess water is essential to prevent further reaction of acetone with hydroxylamine, thereby directing the reaction toward the selective formation of hydroxylamines. The method was applied to the synthesis of paracetamol via acetylation of *N*‐phenylhydroxylamine followed by a 1,5‐rearrangement (Scheme 21a) [89]. In a complementary light‐driven strategy, Y. Sun and coworkers demonstrated that fine Pt nanocrystals (Pt NCs) supported on silica nanospheres (SiO_x_ NSs) catalyze the selective hydrogenation of nitroarenes under visible‐light irradiation (400‐750 nm) at room temperature. Importantly, the optimized photochemical conditions preserve even highly sensitive reducible iodo and aldehyde functionalities. The authors suggested that photoillumination exerts two key effects: i) it lowers the apparent oxidation state of Pt NCs, decreasing the electronegativity of surface Pt atoms and generating high‐energy hot electrons and ii) it lowers the activation energy of the rate‐determining step, as these hot electrons weaken the N‐O bond in surface‐adsorbed Ph‐N(OH)_2_* facilitating OH dissociation to form Ph‐NHOH. Together, these effects synergistically promote cleavage of the N‐O bond and kinetically favored desorption of Ph‐NHOH, preventing over‐reduction to Ph‐NH_2_. Under dark conditions, surface modification with triethanolamine (TEA) similarly tunes the Pt electronic structure, minimizing readsorption of *N*‐arylhydroxylamine derivatives **84** and enhancing selectivity (Scheme 21b) [90].

In visible‐light‐driven photochemical reductions, selective *N*‐arylhydroxylamine formation is achieved through photoexcitation of nitroarenes or activation of hydrogen‐donor reagents. The excited nitroarene species, typically in a triplet (π,π*) or (n,π*) state, can undergo hydrogen atom transfer (HAT) or proton‐coupled electron transfer (PCET) with suitable donors. Metal‐free systems exploit the i) intrinsic photophysical properties of nitroarenes, ii) electron‐donor/acceptor interactions, or iii) functionalized photocatalytic supports to facilitate selective reduction. In metal‐based systems, light irradiation generates high‐energy electrons or transient hot states that modulate the catalyst's electronic properties, weaken the N‐O bond, and promote kinetically favored desorption of hydroxylamine intermediates.

## 3 Synthetic Applications of Monosubstituted *N*‐arylhydroxylamines

This section delineates efficient synthetic approaches that provide an adaptable platform for the generation of heterocyclic libraries from monosubstituted *N*‐arylhydroxylamine precursors, with a special focus on reaction pathway insights. It addresses i) how electronic and steric characteristics of (organo)catalytic systems dictate diastereo‐ and enantioselectivity, ii) the predominant reaction types across diverse methodologies and iii) the key mechanistic steps that guide the formation of complex heterocycles of significant biological potential.

Across all approaches, key mechanistic aspects include: i) the dual reactivity of the *N*‐arylhydroxylamine moiety, which can act as a nucleophile or undergo in situ redox processes, ii) catalytic activation of either the hydroxylamine or the coupling partner through coordination or electrophilic/nucleophilic activation and fine tuning, lowering energy barriers, iii) in situ generation of reactive intermediates (e.g. imines, nitrones) from the hydroxylamine and iv) stereoelectronic control imposed by the catalyst during cycloaddition or nucleophilic attack, ensuring high regio‐ and stereoselectivity through modulation of electronic effects and steric accessibility. While these principles are broadly conserved, certain transformations involve distinct reactive intermediates, rearrangement processes, or solvent/additive effects that modulate the cyclization pathway and selectivity.

**SCHEME 22:**
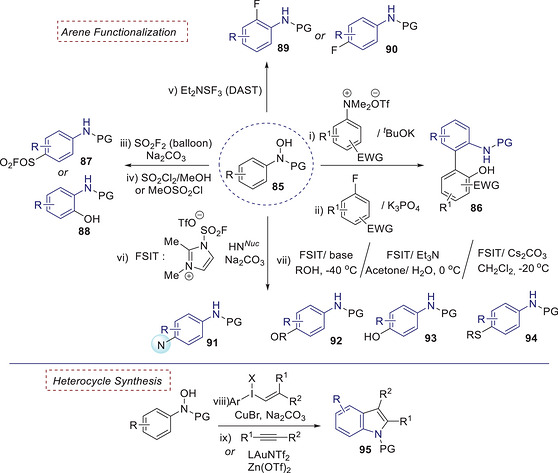
Representative synthetic applications of *N*‐aryl *N*‐protected hydroxylamines **86** via arene functionalization (i–vii) [91, 92, 93, 94, 95, 96, 97] and heterocycle synthesis (viii, ix) [98, 99].

With respect to the class of *N*‐aryl‐*N*‐protected hydroxylamines **85**, notably serving as versatile precursors, representative synthetic applications are concisely summarized in the following scheme and can be grouped into two distinct and directed reactivity paradigms: arene functionalization and heterocycle synthesis. The arene‐functionalization paradigm encompasses site‐selective transformations predominantly enabled by rearrangement‐directed processes, including: i‐ii) nucleophilic substitution of arylhydroxylamines to arylammonium salts or electron‐deficient fluorobenzenes via C‐N bond cleavage accompanied by aerobic C–O bond formation, enabling the synthesis of 2‐hydroxy‐2′‐amino‐1,1′‐biaryls **86**, iii) *para*‐selective SO_2_F_2_‐mediated rearrangement of *N*‐arylhydroxylamines to afford *para*‐amino aryl fluorosulfates **87**, iv) cascade [3, 3]‐sigmatropic rearrangement followed by in situ hydrolysis, promoted by in situ‐generated methyl chlorosulfonate, providing efficient access to 2‐aminophenols **88**, v) deoxyfluorination using diethylaminosulfur trifluoride (DAST) to deliver *ortho*‐ or *para*‐fluorinated aniline derivatives **89–90**, vi) fluorosulfuryl imidazolium triflate‐mediated *para*‐selective C‐H amination and azidation, affording structurally diverse 1,4‐diaminoarenes and *para*‐azidoanilides **91** and vii) regioselective umpolung *para*‐C‐H functionalization for the synthesis of *para*‐functionalized anilides **92–94** from arylhydroxylamines and *O‐* and *S*‐nucleophiles in the presence of fluorosulfuryl imidazolium triflates (FSIT) (Scheme 22) [91, 92, 93, 94, 95, 96, 97]. In parallel, the heterocycle‐synthesis paradigm exploits rearrangement‐driven reactivity of *O*‐functionalized *N*‐arylhydroxylamines, proceeding either via viii) copper‐catalyzed *O*‐vinylation with vinyliodonium salts or through ix) cooperative gold‐zinc catalysis in combination with alkyne precursors, to enable concise access to indole scaffolds **95** (Scheme 22) [98, 99].

### 3.1 Applications of Monosubstituted *N*‐Arylhydroxylamines in the Synthesis of Heterocycles

#### 3.1.1 Synthesis of *β*‐lactam Derivatives

**SCHEME 23:**
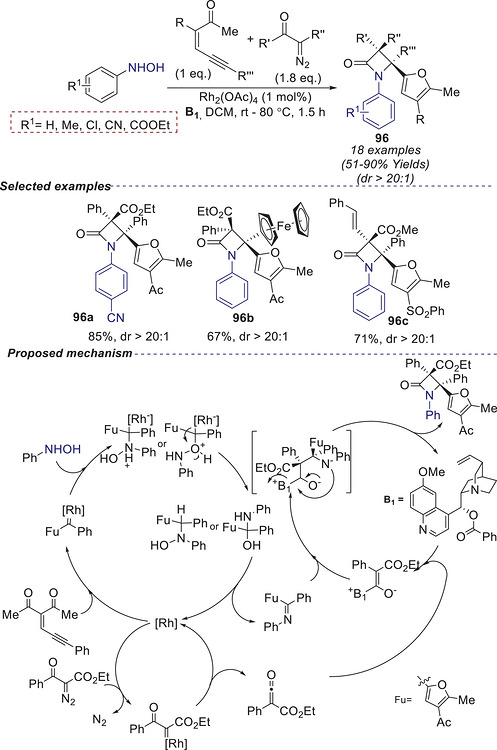
Formation of *β*‐lactams **96** via three‐component reaction of *N*‐arylhydroxylamines, enynones and diazo compounds under combined organo/transition‐metal catalysis [100].

*β*‐Lactams constitute a structural class widely recognized for their importance in medicinal chemistry and natural product synthesis. In this setting, J. Sun and coworkers reported a stereoselective approach to fully substituted *β*‐lactams **96** via a three‐component reaction of *N*‐arylhydroxylamines, enynones, and diazo compounds under organo/transition metal combined catalysis. The transformation proceeds through three distinct catalytic cycles: i) imine formation, ii) Wolff rearrangement and iii) Staudinger cyclization. In the initial step, Rh(II)‐catalyzed activation of enynones generates a 2‐furyl metal‐carbene species, which undergoes a polarity reversal‐type reaction with the *N*‐arylhydroxylamine to form an imine intermediate. Concurrently, the Rh(II)‐catalyzed Wolff rearrangement of diazo compounds provides reactive ketene species. Subsequent Staudinger cyclization promoted by the organocatalyst benzoylquinine (B1) enables nucleophilic attack of the ketene enolate on the imine affording *β*‐lactams with excellent stereoselectivity (dr > 20: 1) (Scheme 23) [100].

**SCHEME 24:**
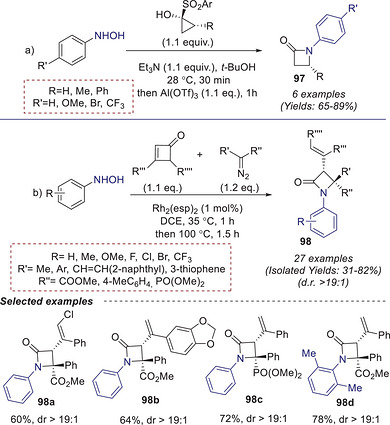
Formation of *β*‐lactams **97** via formal [3+1] cycloaddition of 1‐sulfonylcyclopropanols with *N*‐arylhydroxylamines (a) [101]. Rh(II)‐catalyzed three‐component strategy for the synthesis of *β*‐lactams **98** via the reaction of *N*‐arylhydroxylamines, diazo compounds and cyclobutenones (b) [102].

Also focusing on the *β*‐lactam core, V. N. G. Lindsay and coworkers developed an enantioselective method for the synthesis of 1‐sulfonylcyclopropanols, stable surrogates of cyclopropanone that serve as valuable precursors for the preparation of chiral *β*‐lactams **97**. The transformation proceeds through a formal [3+1] cycloaddition of 1‐sulfonylcyclopropanols with *N*‐arylhydroxylamines under basic conditions, providing *β*‐lactams in high yields and with excellent enantiopurity. Importantly, the reaction is stereospecific, with complete chirality transfer from the cyclopropanol to the *β*‐lactam framework. The crucial ring‐expansion step takes place under Lewis acidic conditions, with aluminium triflate identified as an effective catalytic system. The method is conducted under mild conditions with experimental simplicity and gram‐scale feasibility rendering it suitable for laboratory synthesis. In addition, the protocol shows good compatibility with several *N*‐arylhydroxylamines, enabling access to a range of *β*‐lactam derivatives **97** (Scheme 24a) [101]. More recently, J. Suns’ research group established another three‐component strategy for the synthesis of *β*‐lactams **98**, involving *N*‐arylhydroxylamines, diazo compounds and cyclobutenones under rhodium(II) catalysis. The transformation is proposed to proceed via an in situ‐formed imine, which undergoes a [2+2] cycloaddition with ketenes generated from cyclobutenones. The dirhodium(II) carboxylate complex (Rh_2_(esp)_2_) (1 mol%) was determined as the most effective catalytic system. The method displays a broad substrate scope, high diastereoselectivity (dr > 19:1) and experimental simplicity. Importantly, a variety of functionalized substrates were well‐tolerated highlighting the methodology's generality. Mechanistic studies suggest that the diastereoselectivity arises from stereoelectronic effects during the Staudinger cyclization step. The synthetic utility of the protocol was demonstrated through gram‐scale reactions and post‐synthetic modifications, including base‐mediated isomerization and reduction further emphasizing its practicality for constructing structurally diverse *β*‐lactams (Scheme 24b) [102].

#### 3.1.2 Synthesis of Oxazolidine and Isoxazolidine Derivatives

**SCHEME 25:**
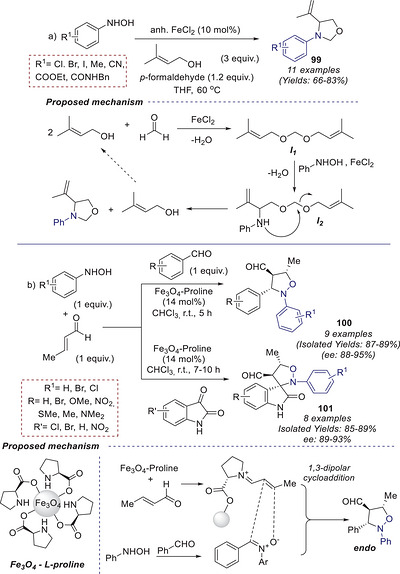
Fe(II)‐catalyzed synthesis of substituted *N‐*aryloxazolidines **99** from *N*‐arylhydroxylamines, allyl alcohols and formaldehyde derivatives (a) [103]. One‐pot, organocatalytic three‐component synthesis of chiral isoxazolidines and spiroisoxazolidines **100–101** using Fe_3_O_4_‐L‐proline (b) [104].

In 2016, R. S. Srivastava disclosed an iron‐catalyzed methodology for the synthesis of substituted *N*‐aryloxazolidines **99** via a dual‐step mechanism involving C‐N bond formation and methylenation. The transformation is achieved by treating *N*‐arylhydroxylamines with allyl alcohols in the presence of formaldehyde or its derivatives which serve as the methylene source. As proposed by the authors, the presence of formaldehyde (or its equivalents) serves as an acetal protecting group for the vulnerable hydroxyl (‐OH) group, thereby facilitating the formation of the new C‐N bond. It is worth mentioning that, anhydrous FeCl_2_ (10 mol%) was identified as the most effective catalyst facilitating both the formation of a methylene acetal intermediate (**I_1_**) and its subsequent allylic amination (**I_2_**). This sequence is followed by an intramolecular nucleophilic substitution resulting in formation of the oxazolidine ring. It was further demonstrated that these oxazolidines can undergo acid‐catalyzed demethylenation to provide *N*‐arylamino alcohols. Overall, the methodology is readily applicable offering good to excellent yields across a range of *N*‐arylhydroxylamines, highlighting its versatility and synthetic utility (Scheme 25a) [103]. In the same year, J. Safaei‐Ghomi and S. Zahedi developed a highly efficient, one‐pot, three‐component protocol for the synthesis of chiral isoxazolidines **100** and spiroisoxazolidines **101** with high diastereoselectivity, utilizing Fe_3_O_4_‐L‐proline nanoparticles (14 mol%) as a magnetic organocatalytic system. The transformation is achieved through a 1,3‐dipolar cycloaddition of in situ generated nitrones with *α,β*‐unsaturated aldehydes or isatins, favoring the formation of the corresponding products with endo configuration and in excellent yields. Important practical features of this catalytic system include short reaction times, high efficiency, good functional group tolerance and the use of a recyclable heterogeneous catalyst (Scheme 25b) [104].

**SCHEME 26:**
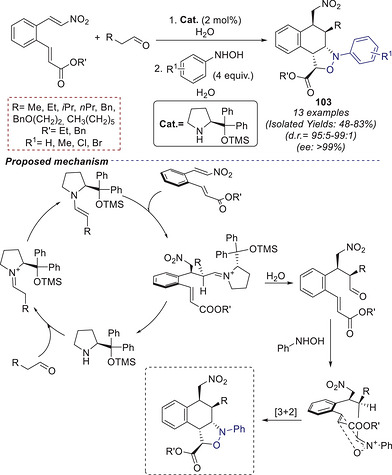
Regio‐ and stereoselective organocatalytic synthesis of tetrahydronaphthalene isoxazolidines **103** [106].

**SCHEME 27:**
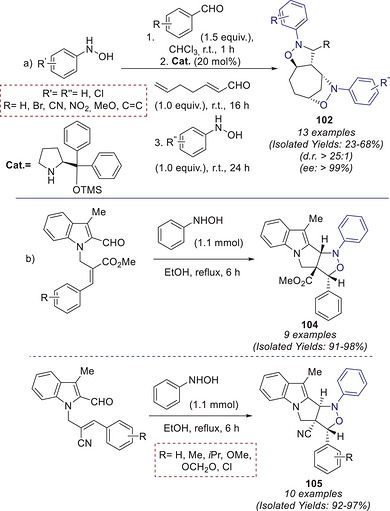
One‐pot organocatalytic cascade synthesis of chiral cycloheptane bis‐isoxazolidine derivatives **102** (a) [105]. Regio‐ and stereoselective synthesis of tetracyclic indolenoisoxazolidines **104–105** using Baylis‐Hillman derivatives, (*Z*)‐methyl 2‐(bromomethyl)‐3‐phenylacrylate precursors (b) [107].

Highly functionalized seven‐membered carbocycles are a common structural motif in numerous natural products as well as synthetic bioactive scaffolds. In this context, A. Córdova and coworkers developed a highly selective cascade synthesis of cycloheptane derivatives **102**, achieving regio‐, chemo‐, diastereo‐ and enantioselectivity. Applying a chiral amine‐based organocatalyst (20 mol%), six new bonds and five stereocenters are assembled with exceptional diastereo‐ and enantioselectivity. The reaction initiates with a chiral‐amine‐catalyzed condensation of saturated aldehydes and *N*‐arylhydroxylamines, generating a nitrone intermediate that undergoes an intramolecular [3+2] cycloaddition with an unsaturated aldehyde. Subsequently, a second nitrone forms and participates in another [3+2] cycloaddition, producing several tricyclic bis‐isoxazolidine derivatives in moderate to good yields (23%–68%, dr > 25:1, *ee*: 98%–99%). This cascade strategy selectively favors seven‐membered rings over six‐membered carbocycles, attributed to steric and substitution effects (Scheme 27a) [105]. Further aiming at the construction of complex heterocycle derivatives, G. Zhong and coworkers developed a highly stereoselective, one‐pot synthesis of chiral isooxazolidine tetrahydronaphthalenes **103**, from *N*‐aryhlydroxylamine precursors using water as a green solvent. Importantly, water serves a dual role: acting as a sustainable solvent and enhancing both reactivity and stereoselectivity, which is attributed to accelerated iminium ion hydrolysis facilitated by Jørgensen's organocatalyst (2 mol%). The reaction is initiated by a tandem Michael addition of the aldehyde to the nitrostyrene, promoted by the pyrrolidine‐based organocatalyst to achieve high stereocontrol. Subsequently, the nitrone is generated in situ, which undergoes an intramolecular [3+2] nitrone‐olefin cycloaddition to construct the fused heterocyclic framework. This protocol efficiently establishes five new stereogenic centers with excellent enantiomeric excesses (*ee*: >99%) and high diastereoselectivities (dr: 95:5 to 99:1) and it is applicable to a broad range of nitroolefin acrylates and aldehydes (Scheme 26) [106].

Alternatively, M. Bakthadoss and coworkers established a regio‐ and stereoselective C‐C bond forming strategy toward tetracyclic indolenoisoxazolidines **104–105** via intramolecular 1,3‐dipolar nitrone cycloaddition. Baylis‐Hillman derivatives, (*Z*)‐methyl 2‐(bromomethyl)‐3‐phenylacrylates, served as versatile precursors for the preparation of the required *N*‐allylated intermediates enabling the construction of complex tetracyclic systems through tandem nitrone formation followed by intramolecular 1,3‐dipolar cycloaddition. This transformation simultaneously generates two new rings and three stereogenic centers including an all‐carbon quaternary center with excellent efficiency (91%–98% yields), under catalyst‐free conditions. Notably, the protocol was also proved effective for nitrile‐functionalized substrates, thereby broadening its synthetic scope (Scheme 27b) [107].

**SCHEME 28:**
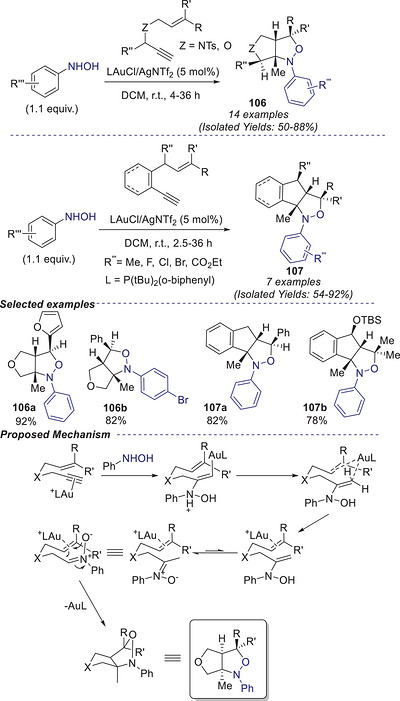
Synthesis of *N,O*‐heterocyclic compounds **106–107** through [2+2+1] annulation of 1,6‐enynes with *N*‐arylhydroxylamines under gold(I) catalysis [108].

Focusing on the construction of diverse *N,O*‐heterocyclic compounds, R.‐S. Liu and coworkers highlighted the [2+2+1] annulation of 1,6‐enynes with *N*‐AHAs under gold(I) catalysis. Remarkably, this protocol features a unique mode of reactivity proceeding via an *N*‐attack at the *π*‐alkyne in contrast to previous gold‐catalyzed processes where *O*‐attack predominates. The key step is proposed to involve the formation of a nitrone intermediate through *N*‐coordination which enables access to functionalized *N,O*‐heterocycles **106–107**. Upon optimization of the reaction conditions it was established that: 1) electron‐rich and bulky gold(I) catalysts (LAuCl L = P‐(*t*Bu)_2_(*o*‐biphenyl)) (5 mol%) more efficiently promoted protodeauration enabling higher yields, 2) alkene‐ and benzene‐bridged 1,6‐enynes were well‐tolerated undergoing smooth annulation, 3) electron‐rich alkenes exhibited decreased reactivity, consistent with nitrone‐alkene cycloadditions and 4) the basicity of the hydroxylamine was critical with less basic derivatives providing superior yields in shorter times. Of particular significance, this transformation exhibits several distinctive features: A) The *N*‐attack of *N*‐hydroxyaniline on the π‐alkyne occurs preferentially over the corresponding *O*‐attack reflecting the higher nucleophilicity of amines compared to alcohols, B) The *N*‐attack pathway is effective for aryl‐substituted alkynes with strong ammonium acidity to enable protodeauration, C) Allyl propargyl ethers undergo exclusive *N*‐attack, with protodeauration facilitated by alkene coordination to the gold center, which directs the observed chemoselectivity. Regarding the mechanism the authors proposed the following sequence: 1) initial *N*‐attack of *N*‐hydroxyaniline on the *π*‐alkyne generates an alkenyl‐gold intermediate, 2) protodeauration occurs through protonation at the Au‐C bond, affording a cationic species, 3) alkene coordination to gold compensates for the destabilization from Au‐C bond cleavage, generating a stabilized intermediate, 4) tautomerization of this species leads to nitrone intermediate, 5) the nitrone moiety featuring a high‐lying HOMO, undergoes stereospecific [3+2] dipolar cycloaddition with Au(I)‐π‐alkene species, whose LUMO is suitably aligned to promote the process with high stereocontrol (Scheme 28) [108].

**SCHEME 29:**
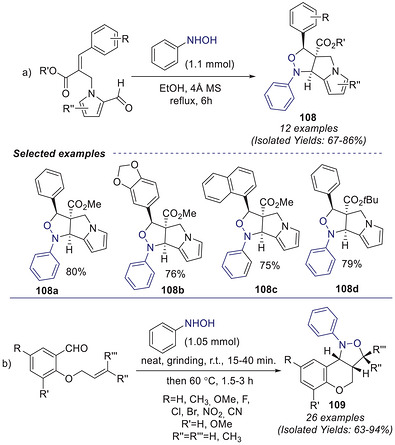
Synthesis of tetrahydro‐*1H*‐isoxazolo[3,4‐*a*]pyrrolizines **108** via the reaction of *N*‐allylated aldehydes with phenyhydroxylamine (a) [109]. Mechanochemical synthesis of *cis*‐fused chromano[4,3‐*c*]isoxazoles **109** from *O*‐allyl salicylaldehyde derivatives and phenylhydroxylamine (b) [110].

A distinct intramolecular nitrone cycloaddition strategy for the synthesis of tetrahydro‐*1H*‐isoxazolo[3,4‐*a*]pyrrolizine derivatives **108** was also reported by M. Bakthadoss and J. Srinivasan. Specifically, using *N*‐allylated aldehydes as precursors an in situ generated nitrone undergoes a [3+2] cycloaddition with phenyhydroxylamine to access tricyclic fused heterocycle derivatives **108**. This methodology is distinguished by its ability to assemble two new rings, introduce three stereocenters, and install a tetrasubstituted carbon center with excellent diastereocontrol affording various highly functionalized heterocycles in good to excellent yields (67%–86%) (Scheme 29a) [109]. Furthermore, M. Banerjee and coworkers developed a catalyst‐ and solvent‐free mechanochemical strategy for the synthesis of *cis*‐fused chromano[*4,3‐c*]isoxazoles **109**. The approach utilizes grinding to promote nitrone formation via the condensation of a variety of *O*‐allyl salicylaldehyde derivatives with phenylhydroxylamine, followed by an intramolecular 1,3‐dipolar cycloaddition to furnish *cis*‐fused tetrahydrochromeno[4,3‐*c*]isoxazole derivatives **109** in high yields (67%–94%). The transformation is proposed to occur in two steps: initial nitrone formation through grinding, followed by cycloaddition accelerated by heating to 60 °C. This methodology stands out for its simplicity, efficiency, short reaction times and high atom economy (Scheme 29b) [110].

#### 3.1.3 Synthesis of Oxazine Derivatives

**SCHEME 30:**
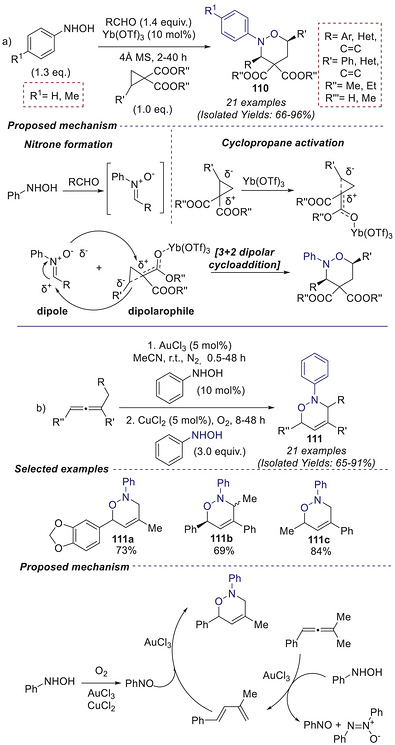
Lewis acid‐promoted three‐component synthesis of 3,6‐dihydro‐*2H*‐1,2‐oxazines **110** from 1,1‐cyclopropanediesters, aldehydes and *N*‐arylhydroxylamines (a) [111]. Synthesis of enantioenriched dihydro‐1,2‐oxazines **111** via a one‐pot asymmetric oxidative nitroso‐Diels–Alder reaction of *N*‐arylhydroxylamines with diene carbamates (b) [112].

Turning toward oxazine‐based heterocycles, the research group of M. A. Kerr reported the synthesis of 3,6‐dihydro‐*2H*‐1,2‐oxazines **110** with high diastereoselectivity and excellent yields based on a distinct three‐component approach involving 1,1‐cyclopropanediesters, aldehydes and *N*‐AHAs under Lewis acid conditions. A crucial feature of this approach is the in situ formation of the nitrone intermediate, avoiding the preparation of potentially unstable nitrone derivatives. The methodology displays a broad substrate scope, incorporating a variety of electron‐rich and electron‐deficient aldehydes as well as diverse cyclopropanediesters. With respect to the reaction mechanism, the process involves the initial condensation of the *N*‐arylhydroxylamine with the aldehyde to form the nitrone intermediate in situ. The cyclopropane is subsequently activated by the Lewis acid Yb(OTf)_3_ (10 mol%) enhancing its reactivity as a dipolarophile. In the final step, the nitrone undergoes [3+2]‐type cycloaddition with the activated cyclopropane providing access to the desired 3,6‐dihydro‐*2H*‐1,2‐oxazine scaffold (Scheme 30a) [111]. As an alternative access route, R. S. Liu and coworkers introduced a distinct and efficient strategy utilizing *N*‐arylhydroxylamines as versatile precursors in combination with allenes which enables the synthesis of the target oxazine scaffolds **111** in the presence of gold and copper catalysts. The catalytic protocol is characterized by excellent yields (65%–91%), broad functional group tolerance and mild reaction conditions demonstrating notable synthetic versatility. From a mechanistic perspective, the transformation is initiated by the isomerization of the allene to the corresponding 1,3‐diene under catalytic amounts of AuCl_3_ (5 mol%) or CuCl (5 mol%) in the presence of hydroxylamine. Noticeably, excess *N*‐arylhydroxylamine reduces the acidity of the gold catalyst, resulting in lower yield. Subsequently, the hydroxylamine is oxidized by the AuCl_3_/CuCl_2_ catalytic system to the corresponding nitroso intermediate, which undergoes a [4+2] cycloaddition process with the conjugated alkene to yield the 3,6‐dihydro‐*2H*‐1,2‐oxazine product (Scheme 30b) [112].

**SCHEME 31:**
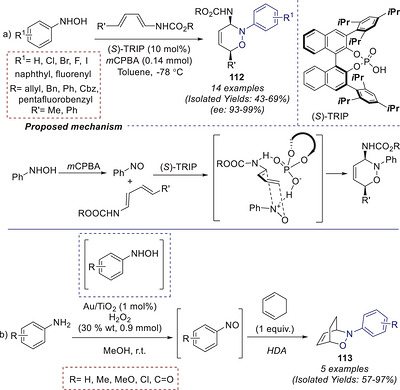
Enantioselective synthesis of dihydro‐1,2‐oxazines **112** via a one‐pot oxidative nitroso‐Diels‐Alder reaction of *N*‐arylhydroxylamines with diene carbamates (a) [113]. Au/TiO_2_‐catalyzed oxidation of arylamines to nitrosoarenes and subsequent hetero Diels‐Alder cycloaddition via *N*‐arylhydroxylamine intermediate (b) [114].

Subsequently, G. Masson and A. Dumoulin disclosed a highly stereoselective method for the synthesis of enantioenriched dihydro‐1,2‐oxazines **112** (*ee*: 93%–99%) via a one‐pot asymmetric oxidative nitroso‐Diels‐Alder reaction of *N*‐arylhydroxylamines with diene carbamates. The transformation relies on the combination of chiral phosphoric acid (10 mol%) and a stoichiometric oxidant (*m*‐CPBA) to generate the nitroso species in situ, achieving excellent regio‐, diastereo‐ and enantioselectivity while suppressing undesired overoxidation or dimerization processes of *N*‐arylhydroxylamines. Importantly, the method demonstrates scalability (up to 20‐fold) and broad functional group compatibility. Furthermore, the nature of the oxidant appears to play a critical role in achieving both reactivity and selectivity, with *m*‐CPBA proving optimal for this transformation. Mechanistic investigations revealed that the chiral phosphoric acid not only dictates enantioselectivity but also reverses the regioselectivity relative to uncatalyzed nitroso‐Diels‐Alder reactions allowing precise control over the reaction outcome (Scheme 31a) [113]. In the same year, I. N. Lykakis and coworkers highlighted the *N*‐arylhydroxylamine moiety as a key intermediate in the Au/TiO_2_‐catalyzed oxidation of arylamines to nitrosoarenes using H_2_O_2_ as the oxidant. Notably, the in situ generated nitrosoarenes efficiently underwent hetero Diels‐Alder reactions with 1,3‐cyclohexadiene, affording the corresponding 1,4‐cycloadducts **113** in high isolated yields (57%–97%). Mechanistic studies indicated that the oxidation of arylamines primarily proceeds via an electron‐transfer (ET) pathway, where the initially formed *N*‐arylhydroxylamine intermediates undergo rapid catalytic oxidation to yield the corresponding nitrosoarenes. The gold nanoparticles were identified as the active species responsible for facilitating both the oxidation and the subsequent cycloaddition processes (Scheme 31b) [114].

**SCHEME 32:**
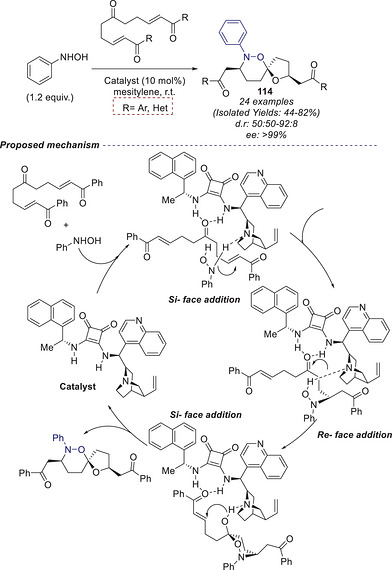
Chiral squaramide‐catalyzed synthesis of spirooxazines **114** from *N*‐substituted hydroxylamines and keto‐bis‐enones via an aza‐Michael/1,2‐addition/oxa‐Michael cascade [115].

In a more recent study, P. Ghorai and P. Gayen introduced an organocatalytic cascade strategy for the enantioselective synthesis of spirooxazines **114** (*ee*: > 99%). The transformation combines *N*‐arylhydroxylamines with keto‐bis‐enones, proceeding through an aza‐Michael/1,2‐addition/oxa‐Michael sequence catalyzed by a chiral bifunctional squaramide (10 mol%). This protocol enables the stereoselective preparation of oxa‐spirooxazines bearing multiple nonadjacent stereocenters. Mechanistic studies suggest the formation of a hemiketal intermediate, which undergoes a stereocontrolled oxa‐Michael addition as the key enantiodetermining step. It is essential to point out that enantioselectivities are consistently excellent (*ee* >99%), while diastereoselectivity is governed by steric and electronic effects, with dr values of up to 92:8. Control experiments confirm that enantioselectivity is established during the final oxa‐Michael step, consistent with a dynamic kinetic asymmetric transformation (DyKAT) pathway. The method tolerates a broad range of enone and hydroxylamine substrates, and its synthetic utility was further demonstrated by scale‐up experiments and post‐functionalization via Suzuki‐Miyaura coupling (Scheme 32) [115].

##### 3.1.3.1 Photoinduced synthesis of oxazine derivatives

**SCHEME 33:**
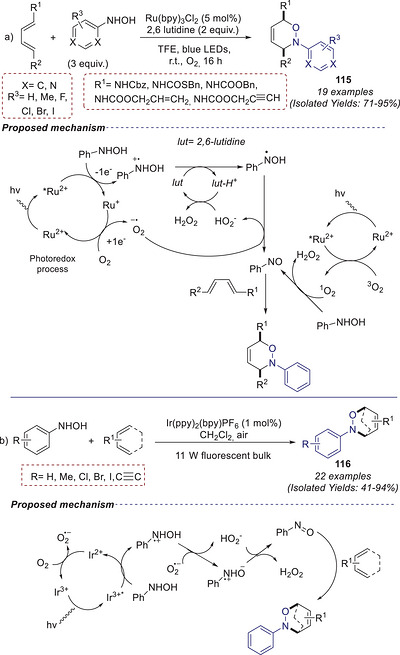
Ru(II)‐mediated visible‐light synthesis of *cis*‐3,6‐dihydro‐1,2‐oxazines **115** via oxidative tandem nitroso Diels‐Alder reaction of *N*‐arylhydroxylamines with conjugated dienes (a) [116]. Visible‐light synthesis of 3,6‐dihydro‐1,2‐oxazines **116** via an Ir(III)‐catalyzed oxidative Diels‐Alder reaction of *N*‐arylhydroxylamines with 1,3‐dienes (b) [117].

A notable contribution was made by G. Masson and coworkers, who demonstrated a visible‐light photoredox‐mediated oxidative tandem nitroso Diels‐Alder reaction of *N*‐AHAs in excess with conjugated dienes. The photocatalytic transformation was conducted in the presence of a photoexcited bis(pyridyl)‐based Ru(II) complex (5 mol%) under blue LED light irradiation in 2,2,2‐trifluoroethanol as the solvent and 2,6‐lutidine as a base within an oxygen atmosphere. As a result a wide range of diverse *cis*‐3,6‐dihydro‐1,2‐oxazines **115** were synthesized in high yields (71%–95%) and with excellent diastereoselectivity. The photocatalytic protocol was effective for various functionalized dienecarbamates and thioenecarbamates, demonstrating exceptional functional group tolerance and compatibility with ethers, thioethers, alkenes and alkynes. Regarding the reaction mechanism, the authors proposed the following steps: i) photoexcited Ru(bpy)_3_^2+^ promotes the oxidation of the *N*‐arylhydroxylamine to generate an ammonium‐hydroxyl radical cation, ii) which upon deprotonation generates a nitrogen‐centered radical species, iii) Subsequent single‐electron transfer with superoxide generates the nitrosoarene, iv) which then undergoes [4+2] cycloaddition with the diene. An alternative pathway may involve direct oxidation by singlet oxygen, emphasizing the dual photochemical reactivity of *N*‐arylhydroxylamines. As a result, this report reveals the potential of *N*‐arylhydroxylamines not only as precursors to reactive nitrogen intermediates but also as photochemically accessible species that can be converted into synthetically valuable organic compounds under mild reaction conditions (Scheme 33a) [116]. In 2025, H. Hou and his research group described a visible‐light photoredox‐catalyzed oxidative Diels‐Alder reaction of *N*‐arylhydroxylamines with 1,3‐dienes, providing access to various 3,6‐dihydro‐1,2‐oxazines **116** in good to excellent isolated yields. The photocatalytic reaction was carried out with 1 mol% of [Ir(ppy)_2_bpy]PF_6_ (1 mol%) in DCM as the optimal solvent and irradiated with an 11 W fluorescent bulb, under an air atmosphere for 48 h. Different substituted *N*‐arylhydroxylamines, various cyclic 1,3‐dienes and open‐chain 1,3‐dienes were successfully applied under the optimized reaction conditions. The proposed photochemical mechanism proceeds through the following sequence: 1) photoexcitation of Ir^3+^ to its excited state Ir^3+*^ under visible‐light irradiation, 2) reductive quenching of Ir^3+*^ by the *N*‐arylhydroxylamine, forming Ir^2+^ and an ammonium radical cation via a single‐electron transfer (SET) process, 3) reoxidation of Ir^2+^ to Ir^3+^ by molecular oxygen, generating superoxide and thereby closing the catalytic cycle, 4) hydrogen‐atom transfer (HAT) from the ammonium radical cation to superoxide producing an iminium ion intermediate and HO_2_^−^ and 5) deprotonation of the hydroxyl group by HO_2_^−^ to form the nitroso intermediate which rapidly undergoes a [4+2] cycloaddition with 1,3‐dienes to yield the corresponding 3,6‐dihydro‐1,2‐oxazine products (Scheme 33b) [117].

#### 3.1.4 Synthesis of *N*‐substituted Pyrroles

**SCHEME 34:**
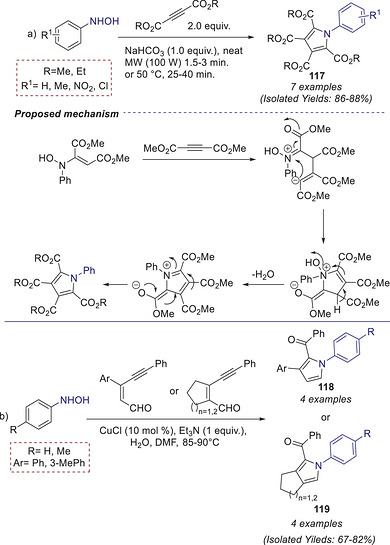
Solvent‐free synthesis of *N*‐substituted pyrrole‐2,3,4,5‐tetracarboxylates **117** via microwave‐assisted reaction of acetylenedicarboxylates with *N*‐arylhydroxylamines (a) [118]. Copper(I)‐catalyzed synthesis of substituted pyrrole and isoindole derivatives **118–119** from *N*‐arylhydroxylamines and 3‐(1‐alkynyl)‐2‐alkene‐1‐als (b) [119].

Exploiting *N*‐AHAs as potential precursors for pyrrole core construction, H. Valizadeh and coworkers presented a simple and efficient solvent‐free strategy for the synthesis of *N*‐substituted pyrrole‐2,3,4,5‐tetracarboxylates **117**, exploring both thermal and microwave‐assisted conditions. The transformation involves the reaction of dimethyl‐ or diethyl acetylenedicarboxylates with *N*‐arylhydroxylamines in the presence of NaHCO_3_ affording highly substituted pyrroles in excellent yields. Notably, the microwave‐assisted protocol significantly reduces reaction times relative to standard thermal conditions at 50°C achieving yields of up to 88%. The proposed mechanism proceeds through an initial Michael addition of the hydroxylamine to the acetylenedicarboxylate giving an enamine‐like intermediate, which undergoes cyclization to a zwitterionic species. Subsequent dehydration gives the pyrrole scaffold. Generally, this methodology offers distinct advantages including shorter reaction times, improved efficiency and enhanced sustainability owing to the absence of solvents, making it an attractive approach to highly functionalized pyrroles (Scheme 34a) [118]. In the next year, J. K. Ray and S. Nandi developed a copper(I)‐catalyzed methodology for the synthesis of highly substituted pyrrole and isoindole derivatives **118–119** from *N*‐arylhydroxylamines. The catalytic transformation involves the reaction of 3‐(1‐alkynyl)‐2‐alkene‐1‐als with *N*‐arylhydroxylamines using CuCl as the catalyst (10 mol%), Et_3_N as the base and DMF as the solvent under thermal conditions. Notably, in the absence of water, the yield was significantly dropped, indicating its essential role in the reaction. Under optimal conditions, the method provides pyrrole and isoindole derivatives in moderate to good yields (77%–82%). With respect to substrate scope, it is noteworthy that the reaction did not proceed when 3‐(4‐methoxyphenyl)‐5‐phenylpent‐2‐en‐4‐ynal was employed. In this case, it was proposed that the extended conjugation possibly stabilizes an unreactive resonance form thereby preventing cyclization (Scheme 34b) [119].

#### 3.1.5 Synthesis of Indole Derivatives

**SCHEME 35:**
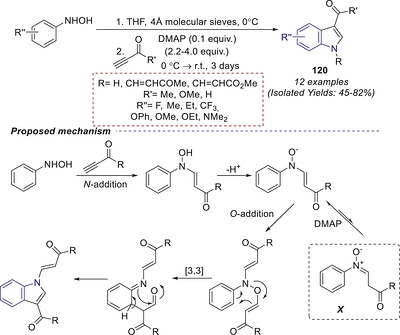
DMAP‐mediated synthesis of polysubstituted indole derivatives **120** from *N*‐arylhydroxylamines and activated terminal alkynes [120].

The standard Fischer indole synthesis, based on the condensation of phenylhydrazine derivatives with carbonyl compounds under acidic conditions, has long been established. More recently, however, *N*‐arylhydroxylamines have emerged as alternative precursors for indole construction. In 1994, M. O. Gray and coworkers reported the first synthesis of polysubstituted indole derivatives **120** employing unprotected *N*‐arylhydroxylamines. The protocol relies on activated terminal alkynes, DMAP as a base, and 4 Å molecular sieves, affording indole derivatives in 52%–78% yields. The reaction proceeds through sequential alkyne additions, initially at nitrogen and subsequently at oxygen, followed by a [3, 3]‐sigmatropic rearrangement and rearomatization. In the absence of DMAP, the reaction shifts toward isoxazoline products via a nitrone intermediate, highlighting the crucial role of the base in controlling the pathway. Moreover, when the hydroxylamine carried a CF_3_ substituent at the 2‐position, the reduced nucleophilicity of nitrogen favored the formation of non‐*N*‐substituted indoles (Scheme 35) [120].

**SCHEME 36:**
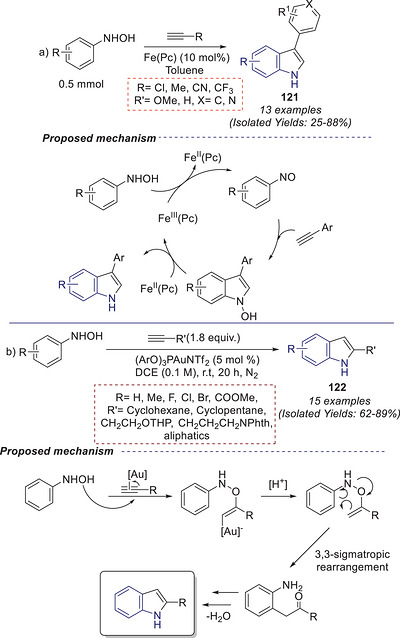
Fe(Pc)‐catalyzed synthesis of 2′‐ and 3′‐substituted indoles **121** from aliphatic alkynes and *N*‐arylhydroxylamines (a) [121]. Gold(I)‐catalyzed synthesis of 2‐alkylindoles **122** via annulation of *N*‐arylhydroxylamines with terminal alkynes (b) [122].

Toward the construction of indole scaffolds, A. A. Lamar and K. M. Nicholas developed a versatile iron‐catalyzed methodology for the synthesis of 3′‐substituted indole derivatives from aliphatic alkynes and *N*‐arylhydroxylamines. This catalytic method enables efficient synthesis of 3‐alkyl‐ and 3‐arylindoles **121** with moderate to excellent yields and excellent regioselectivity using 10 mol% of an iron(II) phthalocyanine (Fe(Pc) complex. The proposed catalytic pathway involves in situ oxidation of the hydroxylamine to generate a nitrosoarene, followed by an ene‐type reaction with the alkyne to form an *N*‐hydroxyindole intermediate, which is subsequently reduced to the corresponding indole. It is important to note that: 1) terminal alkynes exhibit significantly higher reactivity and yield compared to internal alkynes, and 2) the methodology tolerates a broad range of *N*‐arylhydroxylamines as well as diverse aryl alkynes. Mechanistically, the reaction is proposed to occur via Fe^3+^(Pc)‐mediated oxidation of PhNHOH to the nitrosoarene, cyclocondensation with the alkyne to form the *N*‐hydroxyindole, and subsequent reduction by Fe^2+^(Pc) to afford the desired indole product (Scheme 36a) [121]. The same year, L. Zhang and coworkers disclosed a regiospecific synthesis of 2‐alkylindoles **122** via the annulation of *N*‐arylhydroxylamines with several terminal alkynes under inert atmosphere at room temperature. They proposed that a phosphite‐based cationic gold(I) complex promote the formation of *O*‐alkenyl‐*N*‐arylhydroxylamine species through activation of the alkyne toward nucleophilic attack. These key intermediates then undergo a Fischer‐type [3, 3]‐sigmatropic rearrangement, followed by cyclodehydration to afford 2‐alkylindoles exclusively with no formation of 3‐alkyl isomers and in high yields. This methodology combines Markovnikov selectivity, high efficiency and mild reaction conditions providing a practical and regiospecific route to 2‐alkylindoles (Scheme 36b) [122].

**SCHEME 37:**
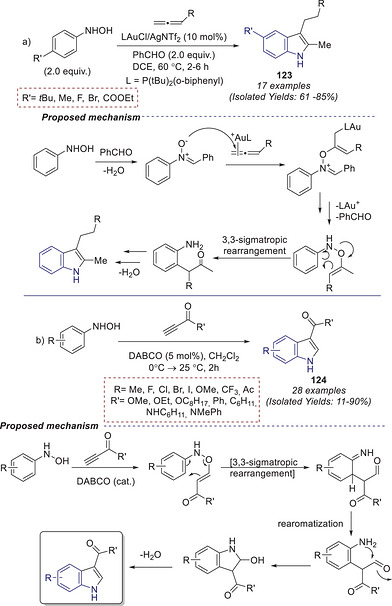
Gold‐catalyzed synthesis of 2,3‐disubstituted indoles **123** from allenes and *N*‐arylhydroxylamines (a) [123]. DABCO‐promoted Fischer‐type synthesis of 3‐substituted indoles **124** from *N*‐arylhydroxylamines and activated terminal alkynes (b) [124].

Also, in the context of indole synthesis, R.‐S. Liu and coworkers reported a gold‐catalyzed protocol for the synthesis of 2,3‐disubstituted indoles **123** from a series of allenes and *N*‐AHAs. The reaction, performed in refluxing DCE with LAuCl/AgNTf_2_ (10 mol %) as the catalytic system afforded the indole products in high yields. The catalytic efficiency was found to depend strongly on the acidity of the gold complex, with less acidic catalysts showing minimal activity. A notable feature of this transformation is that the hydroxylamine condenses with benzaldehyde to form a nitrone, which acts as the key reactive species. Since unactivated allenes were employed the conventional [3+2] nitrone‐allene cycloaddition was suppressed thereby redirecting the pathway toward indole formation. Regarding the mechanism, the authors proposed the following sequence: 1) condensation of *N*‐arylhydroxylamine with benzaldehyde generates the nitrone species, 2) nucleophilic attack of the nitrone oxygen on the gold‐activated allene, 3) hydrolysis of the resulting iminium species generates an enol ether intermediate with concurrent release of benzaldehyde moiety and regeneration of the LAu^+^ species, 4) a subsequent [3, 3]‐sigmatropic rearrangement of the enol ether forms an aniline intermediate bearing a tethered ketone, 5) intramolecular cyclization of this intermediate then delivers the 2,3‐disubstituted indole derivatives (Scheme 37a) [123]. The most recent advancement in the synthesis of 3‐substituted indoles **124** was reported by F. García‐Tellado and coworkers. This methodology utilizes *N*‐arylhydroxylamines as alternative candidates to classical arylhydrazines in a Fischer‐type indole synthesis, combined with activated terminal alkynes to access poly‐substituted indoles bearing an electron‐withdrawing group at the C‐3 position. The key step involves a DABCO‐catalyzed addition of the hydroxylamine to the alkyne generating an *N*‐oxyenamine intermediate that undergoes a [3, 3]‐sigmatropic rearrangement followed by cyclization to form the indole core. The protocol exemplifies step economy by avoiding *N*‐protection/deprotection sequences while enabling the incorporation of a broad array of functionalized nitroarenes, providing access to structurally diverse indoles with varied substitution patterns and topologies. Under optimized conditions consisting of 5 mol% DABCO and temperatures between 0 °C and 25 °C polysubstituted indoles were obtained in high yields under mild, metal‐free and acid‐free conditions (Scheme 37b) [124].

#### 3.1.6 Synthesis of Miscellaneous Heterocycle Derivatives

**SCHEME 38:**
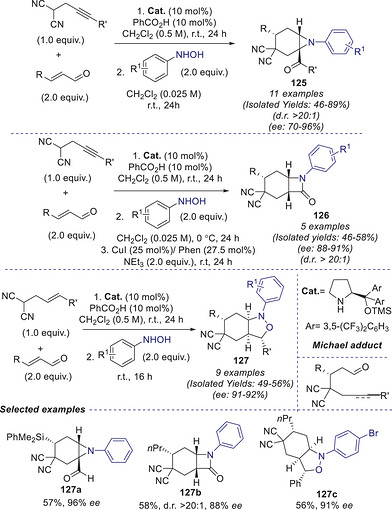
Synthesis of aziridine carbonyls, *β*‐lactams and octahydrobenzo[*c*]isoxazoles **125–127** from *N*‐arylhydroxylamines, aliphatic *α,β*‐unsaturated aldehydes and substituted malononitriles [125].

A versatile synthetic methodology was reported in 2011 by K. A. Jørgensen and coworkers utilizing *N*‐AHAs along with aliphatic *α,β*‐unsaturated aldehydes and substituted malononitriles to access a variety of heterocycles, including aziridine carbonyls, *β‐*lactams, and octahydrobenzo[*c*]isoxazoles **125–127** with high enantiomeric excess and excellent diastereoselectivity. The crucial step in this catalytic procedure is the formation of a Michael adduct, promoted by the combination of (*S*)‐2‐[bis(3,5‐bistrifluoromethylphenyl)trimethylsilyloxymethyl]pyrrolidine (10 mol%) and benzoic acid (10 mol%) which serves as the pivotal intermediate for divergent product formation. As a result: 1) when the malononitrile bears an alkyne functionality, treatment with the hydroxylamine at room temperature affords the corresponding aziridine, 2) with an alkene‐functionalized malononitrile, the process leads to the formation of an octahydrobenzo[c]isoxazole and 3) in the presence of copper(I) iodide, phenanthroline, and triethylamine, the alkyne‐functionalized malononitrile undergoes a Kinugasa reaction to furnish a bicyclic *β*‐lactam. Mechanistic investigations suggest that all three pathways occur via a 1,3‐dipolar cycloaddition, initiated by the in situ formation of a nitrone intermediate upon addition of the hydroxylamine (Scheme 38) [125].

**SCHEME 39:**
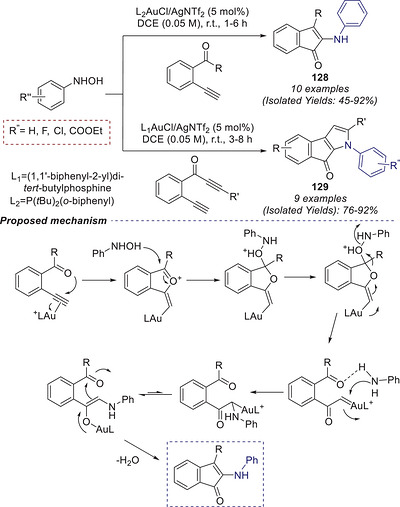
Gold‐catalyzed oxidative transformation of 2‐ketonyl‐1‐ethynylbenzenes with *N*‐arylhydroxylamines for the synthesis of 2‐aminoindenones **128–129** [126].

In 2016, R.‐S. Liu and coworkers envisioned a gold‐catalyzed oxidative transformation of 2‐ketonyl‐1‐ethynylbenzenes with *N*‐arylhydroxylamines affording 2‐aminoindenone derivatives **128** with remarkable efficiency. In contrast to established gold‐catalyzed oxidations which generally proceed through direct attack of oxidants on π‐alkynes, this pathway is strongly supported by ^18^O‐labeling experiments and reveals a distinct oxoamination process. It is worth noting that a series of alkynoyl‐derived substrates efficiently directed the reaction toward the corresponding indeno[2,1‐*b*]pyrrol‐8(*1H*)‐ones **129** in high yields at room temperature, using a LAuCl/AgNTf_2_ catalytic system (5 mol%). Regarding the mechanism, the authors proposed the following sequence: 1) intramolecular nucleophilic attack of the tethered ketone on the gold‐activated π‐alkyne initiates a rapid 5‐exo‐dig cyclization generating an oxonium intermediate, 2) nucleophilic attack by *N*‐arylhydroxylamine converts this species into an *O*‐bound adduct, 3) a 1,2‐proton transfer takes place with protonation occurring preferentially at nitrogen due to its greater basicity producing an ammonium species, 4) cleavage of the N‐O bond then occurs releasing an *α*‐oxo gold carbene, a key intermediate that directs the formation of the desired 2‐aminoindenone derivatives. Isotopic labeling experiments demonstrated that the carbonyl oxygen atom in the resulting indenone originates exclusively from the ketone moiety of the substrate, thereby ruling out the involvement of a nitrone intermediate (Scheme 39) [126].

**SCHEME 40:**
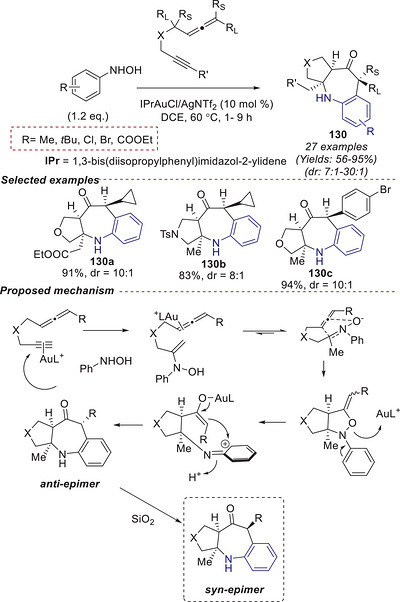
Gold‐catalyzed synthesis of benzo[*b*]azepin‐4‐ones **130** from 6‐allenyl‐1‐ynes and *N*‐arylhydroxylamines [127].

In the following year, R.‐S. Liu and coworkers developed a gold‐catalyzed protocol for the synthesis of benzo[*b*]azepin‐4‐ones **130** starting from 6‐allenyl‐1‐ynes and *N*‐arylhydroxylamines. In this transformation, the substrates first generate nitrone intermediates that undergo intramolecular cyclization to give benzoazepin‐4‐one derivatives which subsequently convert into the desired products. Optimal reactivity was achieved using a LAuCl/AgNTf_2_ catalytic system (10 mol %) in dichloroethane at 60°C. The method tolerates a broad substrate scope, including a variety of *O*‐ and *N*‐linked alkyl‐substituted allenes as well as diverse *N*‐arylhydroxylamines, affording products in high yields and with good diastereoselectivity. A particularly intriguing feature of this chemistry is the preferential isolation of the kinetically disfavored *syn*‐epimer. Mechanistic studies suggest that cyclization is anti‐selective, however the initially formed *anti*‐epimer undergoes epimerization to the *syn*‐form upon treatment with silica. Such chemoselectivity and antiselectivity is rationalized by a postulated gold‐enolate intermediate bearing an anilinium moiety, whose preferred conformation dictates both the chemoselectivity and stereoselection of the benzoazepin‐4‐one products (Scheme 40) [127].

### 3.2 Synthesis of Non‐Ηeterocyclic Compounds From Monosubstituted *N*‐arylhydroxylamines

#### 3.2.1 Synthesis of Nitrones/Cupferrons

**SCHEME 41:**
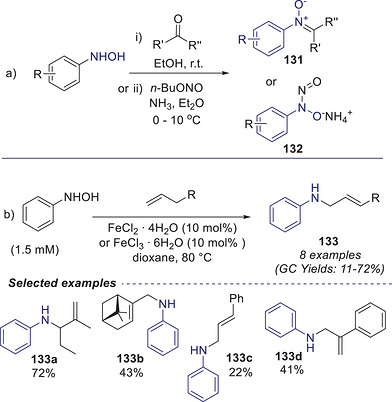
Synthesis of nitrones **131** via condensation of *N*‐arylhydroxylamines with carbonyl compounds (ai) [128]. Synthesis of *N*‐nitroso‐*N*‐phenylhydroxylamines (Cupferrons) **132** from *N*‐arylhydroxylamines via reaction with n‐butyl nitrate (aii) [129, 130]. Iron‐catalyzed regioselective allylic amination of alkenes (b) [131].

Within their diverse transformations*, N*‐arylhydroxylamines are well‐established to serve as versatile precursors for the synthesis of nitrones **131** via condensation with aldehydes or ketones in alcoholic media. The resulting nitrones represent a valuable class of 1,3‐dipoles widely exploited in heterocyclic synthesis through cycloaddition reactions with alkenes and alkynes to yield isoxazolidine and isoxazoline derivatives (Scheme 41ai) [128]. Another noteworthy class of derivatives prepared from *N*‐arylhydroxylamines is *N*‐nitroso‐*N*‐phenylhydroxylamines (Cupferrons), obtained via their reaction with an NO^+^ source. The most widely applied procedure, reported by C. S. Marvel, involved *n*‐butyl nitrate as the NO^+^ donor in the presence of anhydrous ammonia in diethyl ether at 0°C. By applying this synthetic methodology, a series of Cupferrons **132** bearing diverse substituents on the aromatic ring have been successfully synthesized (Scheme 41aii) [129, 130].

#### 3.2.2 Amination Processes

Also shifting toward nonheterocyclic synthetic pathways, K. M. Nicholas and coworker reported an iron‐catalyzed protocol for the regioselective allylic amination of alkenes using phenylhydroxylamine as the aminating reagent. Specifically, both Fe(II) and Fe(III) salts (10 mol%) displayed high efficacy, producing *N*‐phenyl‐*N*‐allylamines **133** in moderate to good yields with excellent regioselectivity, particularly for trisubstituted and 1,1‐disubstituted alkenes. Unlike Mo‐catalyzed systems, this process circumvent the generation of free nitrosobenzene intermediates, thereby suppressing the formation of undesired byproducts such as aniline and azobenzene while still retaining the characteristic ene‐type regioselectivity. Mechanistic evidence points to a Fe‐coordinated species rather than free nitrosobenzene as the active intermediate. The comparable reactivity of Fe(II) and Fe(III) salts suggests either the participation of a common catalytic species or a redox cycling mechanism between the two oxidation states. Overall, this methodology exploits the advantages of iron catalysis including low cost, wide availability and enhanced selectivity over traditional stoichiometric allylic amination methods (Scheme 41b) [131].

**SCHEME 42:**
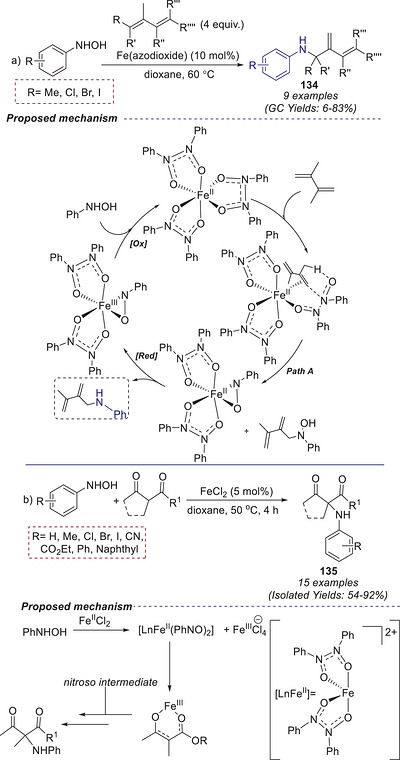
C‐H amination of 1,3‐dienes with *N*‐arylhydroxylamines catalyzed by an iron azobenzene dioxide complex (a) [132]. Fe‐catalyzed *α*‐amination of 1,3‐dicarbonyl compounds to access *α*‐amino carbonyl derivatives **135** (b) [133].

Alternatively, R. S. Srivastava and S. Murru reported an iron‐catalyzed strategy for the selective C‐H amination of 1,3‐dienes using *N*‐AHAs as nitrogen sources, with an iron azobenzene dioxide complex (10 mol%) identified as the optimal catalyst for achieving high selectivity. The reaction takes place via in situ formation of nitrosoarenes which react with dienes to yield dienyl allylamines **134** as the major products alongside minor hetero‐Diels‐Alder (HDA) byproducts. Mechanistically, the process involves the following sequence: 1) oxidation of *N*‐arylhydroxylamine by the iron catalyst to generate an activated nitrosoarene‐Fe complex, 2) coordination of the 1,3‐diene to the iron center, forming either an η2 or η4 complex depending on the substitution pattern and conformation of the diene, 3) allylic C‐H amination versus HDA pathway: the η2 diene complex directs selective allylic amination via formation of a dienyl *N*‐hydroxyallylamine‐Fe intermediate (path A), whereas the η4 diene complex favors formation of HDA adducts (path B), 4) reduction/deoxygenation: the dienyl *N*‐hydroxyallylamine intermediate undergoes Fe‐mediated reduction to yield the target dienyl allylamine, regenerating the Fe(III) catalytic species. The η2 coordination is particularly favored for acyclic 2,3‐substituted dienes, which adopt an s‐cis conformation, stabilizing the iron complex and directing the reaction toward selective allylic amination. Hammett analysis further supports partial negative charge development on the nitroso nitrogen during the process, consistent with the iron‐stabilized intermediates (Scheme 42a) [132]. In parallel, the same research group reported a direct Fe‐catalyzed *α*‐amination of 1,3‐dicarbonyl compounds using arylhydroxylamines as the aminating agents. This protocol provides rapid access to α‐amino carbonyl derivatives **135** in high yields with excellent *N*‐selectivity. Mechanistic studies revealed a dual role of the iron catalyst: Fe(II) facilitates the generation of electrophilic nitroso intermediates from arylhydroxylamines, while Fe(III) acts as a Lewis acid to activate the 1,3‐dicarbonyl nucleophiles. The method displays broad substrate scope, encompassing *β*‐ketoesters, 1,3‐diketones, ethyl 2‐oxocyclopentane carboxylate and 3‐methyl‐2‐oxindole, the latter representing a scaffold of notable biological relevance In contrast to 1,3‐dicarbonyl substrates, 2‐methylcyclohexanone gave the α‐aminated product in only moderate yield (54%), reflecting the superior activation of 1,3‐dicarbonyl derivatives under the reaction conditions (Scheme 42b) [133].

**SCHEME 43:**
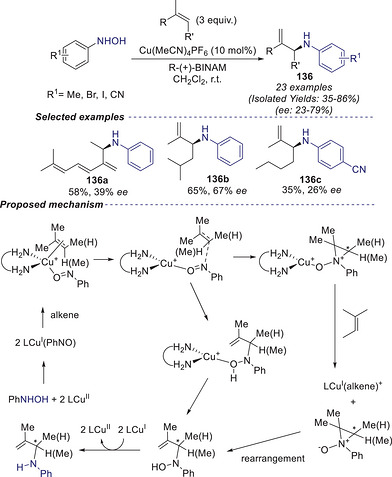
Copper‐catalyzed asymmetric allylic C‐H amination of alkenes with *N*‐arylhydroxylamines [134].

A few years later, R. S. Srivastava and coworkers reported a copper‐catalyzed asymmetric allylic C‐H amination of alkenes exploring *N*‐arylhydroxylamines as the aminating agents. The transformation utilizes Cu(MeCN)_4_PF_6_ (10 mol%) as a precatalyst in combination with the chiral diamine ligand R‐(+)‐BINAM, affording the *N*‐allyl arylamines **136** in high yields (up to 86%) and with good enantioselectivities (up to 79%). Mechanistic investigations revealed the formation of a key, (BINAM)Cu(PhNO)(η^2^ ‐alkene)^+^ intermediate which controls the stereochemical outcome of the reaction. The proposed catalytic cycle proceeds as follows: i) in situ generation of the chiral catalyst through coordination of R‐(+)‐BINAM to Cu(MeCN)_4_PF_6_, yielding the active (BINAM)Cu^+^ species, ii) simultaneous coordination of the alkene and the nitrosoarene (PhNO) (generated in situ from the corresponding *N*‐arylhydroxylamine) to the copper center, forming the pivotal (BINAM)Cu(PhNO)(η^2^‐alkene)^+^ complex, iii) C‐N bond formation via a concerted, asynchronous transition state, wherein the O‐N moiety of the coordinated nitroso species inserts into the allylic C‐H bond of the bound alkene, representing the stereodetermining step, iv) this transition state leads to either a Cu‐bound *N*‐allyl arylhydroxylamine or a Cu‐coordinated aziridine *N*‐oxide intermediate, v) subsequent H‐shift and ring‐opening rearrangement produce the *N*‐allyl arylhydroxylamine species, and vi) reduction of the *N*‐allyl arylhydroxylamine furnishes the desired *N*‐allyl arylamine, accompanied by reoxidation of the copper center to regenerate the active (BINAM)Cu^+^ catalyst, thereby completing the Cu(I)/Cu(II) catalytic cycle (Scheme 43) [134].

#### 3.2.3 P‐N Bond Formation

**SCHEME 44:**
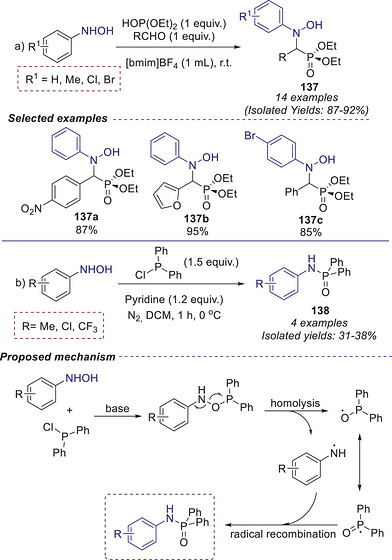
Three‐component synthesis of *α*‐hydroxylaminophosphonates **137** from aldehydes, hydroxylamines and phosphites using [(bmim)BF_4_] (a) [135]. Catalyst‐free synthesis of phosphinic amides **138** via the reaction of *N*‐arylhydroxylamines with chlorophosphines (b) [136].

With respect to P‐N bond formation, a one‐pot, three‐component synthesis of *α*‐hydroxylamino phosphonates was reported by J. S. Yadav and coworkers using an ionic liquid as the reaction medium. In this protocol, aldehydes, hydroxylamines and phosphites engage effectively to afford the target *α*‐hydroxylamino phosphonates. It is worth mentioning that the 1‐butyl‐3‐methylimidazolium tetrafluoroborate ionic liquid [(bmim)BF_4_] not only accelerates the reaction and enhances selectivity but also provides a greener alternative to conventional solvents. The transformation takes place under mild, catalyst‐free conditions, minimizing waste generation. The methodology exhibits a broad substrate scope, tolerating a variety of aldehydes and phosphites, and the ionic liquid can be recycled, further improving the sustainability of the process, offering an environmentally friendly approach to synthesizing valuable organophosphorus compounds with potential applications in pharmaceuticals and agrochemicals (Scheme 44a) [135]. In 2023, S. Xu and coworkers developed an efficient, catalyst‐free method for the synthesis of phosphinic amides via the reaction of *N*‐arylhydroxylamines with chlorophosphines under basic conditions. The transformation proceeds through a P(III) to P(V) rearrangement, circumventing the generation of phosphine oxide byproducts. Mechanistically, the reaction involves the initial formation of a phosphorus‐containing intermediate (R_2_N‐O‐PR_2_), followed by homolysis of the N‐O bond and radical recombination which preferentially occurs at the phosphorus center due to spin density distribution. Optimization studies identified pyridine as an effective base and dichloromethane at 0 °C as the optimal solvent, providing good yields. The methodology demonstrates a broad substrate scope tolerating a variety of *N*‐arylhydroxylamines and chlorophosphines. Furthermore, radical trapping experiments and computational analyses confirmed the involvement of phosphorus‐centered radicals, and the study also highlights potential applications of these radicals in novel bond‐forming reactions, offering a sustainable and atom‐economical route to phosphinic amides (Scheme 44b) [136].

#### 3.2.4 Amide Synthesis

**SCHEME 45:**
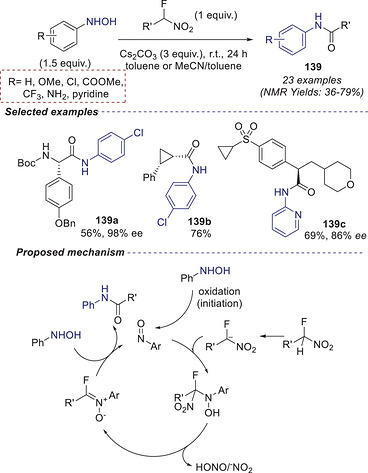
Umpolung amide synthesis (UmAS) via Brønsted base‐promoted reaction between *α*‐fluoronitroalkanes and *N*‐arylhydroxylamines. [137].

In 2022, J. N. Johnston's research group reported a novel umpolung amide synthesis (UmAS) for the direct preparation of *N*‐arylamides **139**, addressing the epimerization issues associated with classical amide‐synthetic methodologies. Their strategy relies on a Brønsted base‐promoted reaction between *α*‐fluoronitroalkanes and *N*‐arylhydroxylamines thereby avoiding external activating agents and proceeding through a redox‐mediated pathway. Under optimal conditions using cesium carbonate (Cs_2_CO_3_) at room temperature, the reaction afforded up to 76% yield of the desired amides with excellent chemoselectivity and complete stereochemical control, under mild and catalyst‐free conditions. Mechanistic investigations support a stepwise sequence: 1) the *N*‐arylhydroxylamine is first oxidized to the corresponding aryl nitroso species, 2) this nitroso electrophile reacts with an *α*‐fluoronitronate anion to generate a haloamino nitroalkane (HANA), 3) subsequent fragmentation of the HANA intermediate produces a key fluoronitrone species and 4) a distinct *N*‐arylhydroxylamine acts as a reductant facilitating amide synthesis. Crucially, the formation of hydroxamic acid is suppressed while the dual engagement of nitroso and hydroxylamine species reflects the cooperative mechanistic pathway (Scheme 45) [137].

#### 3.2.5 Desulfurdioxidative *N*‐*N* Coupling

**SCHEME 46:**
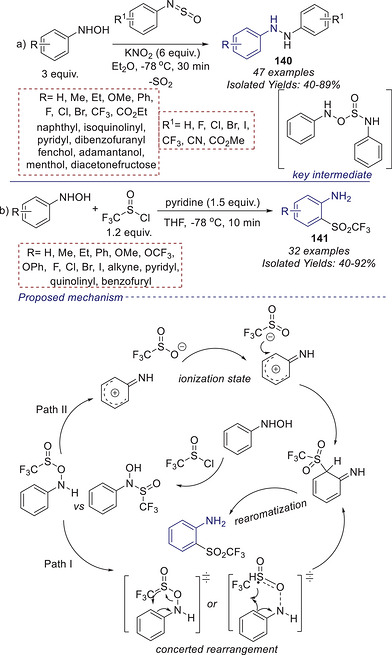
Metal‐free *ortho*‐trifluoromethanesulfonylation of *N‐*arylhydroxylamines for regioselective *ortho*‐functionalized anilines **140** (a) [138]. Metal free desulfurdioxidative N‐N coupling of *N*‐arylhydroxylamines and *N*‐sulfinylanilines for the synthesis of unsymmetrical *N,N′‐*diarylhydrazines **141** (b) [139].

Z.‐X. Yu, H. Gao and coworkers outlined a highly efficient and chemoselective metal‐free strategy for the divergent construction of unsymmetrical hydrazines **140** via an unprecedented intermolecular desulfurdioxidative N‐N coupling process. This protocol utilizes readily accessible *N*‐arylhydroxylamines and *N*‐sulfinylanilines, affording structurally diverse *N,N′‐*diarylhydrazines in good to excellent yields under remarkably mild conditions with broad functional‐group tolerance. Mechanistic investigations, supported by density functional theory (DFT) calculations, revealed that the reaction proceeds through in situ formation of an *O*‐sulfenylated arylhydroxylamine intermediate promoted by KNO_2_. This intermediate undergoes a stepwise diradical pathway involving a rare retro‐[2π+2σ] cycloaddition, accompanied by extrusion of SO_2_ to form the N‐N bond. Notably, σ‐bond homolysis during the retro‐[2π+2σ] process was identified as the rate‐determining step. The method further demonstrates excellent chemoselectivity and synthetic utility, enabling efficient conversion of *N*‐AHAs bearing natural product‐ and pharmaceutical‐relevant motifs, such as fenchol, adamantanol, menthol and diacetonefructose, into the corresponding *N,N′‐*diarylhydrazine derivatives (Scheme 46a) [138].

#### 3.2.6 *ortho*‐Selective Trifluoromethanesulfonylation

X. Qi, H. Gao and coworkers developed a metal‐ and oxidant‐free protocol for the synthesis of synthetically versatile *ortho*‐trifluoromethanesulfonylated anilines **141** from *N*‐arylhydroxylamines and trifluoromethanesulfinic chloride. The transformation proceeds rapidly under basic conditions at low temperature, yielding the desired products in good yields with excellent *ortho*‐selectivity. Two plausible mechanistic pathways were proposed: **I)** *N*‐arylhydroxylamine acts as a nucleophile toward trifluoromethanesulfinic chloride to generate an *O*‐sulfinylated intermediate, which undergoes a concerted [2, 3]‐sigmatropic rearrangement or an open‐shell radical‐type concerted rearrangement to form a dearomatized intermediate, followed by rearomatization to afford the final product. **II)** Alternatively, the *O*‐sulfinylated intermediate may undergo heterolytic N‐O bond cleavage to produce an ion‐pair species, which recombines through nucleophilic attack of the trifluoromethanesulfinate anion to generate the corresponding dearomatized intermediate prior to rearomatization. Control experiments and DFT calculations supported involvement of N‐O bond cleavage and a favorable concerted [2, 3]‐sigmatropic rearrangement pathway, with selective *O*‐site sulfinylation playing a crucial role in directing *ortho*‐functionalization. The method exhibits broad substrate scope, including heteroaryl hydroxylamines such as pyridyl, quinolinyl and benzofuryl derivatives, and the aniline products can be prepared on gram scale and readily diversified into a range of functional molecules (Scheme 46b) [139].

## 4 Summary and Outlook

Since now, electron‐poor *O*‐acyl and *O*‐aryl hydroxylamines, have emerged as versatile precursors for nitrogen‐centered radicals in visible‐light photochemistry. These hydroxylamine derivatives undergo single‐electron transfer (SET) with a photoexcited photocatalyst to promote N‐O bond homolysis, generating nitrogen radicals that engage in a wide range of organic transformations. These studies establish the key principles governing the photochemistry of hydroxylamine derivatives and highlighted their utility as radical precursors for constructing nitrogen‐containing scaffolds [140]. In contrast, *N*‐arylhydroxylamines remain largely underexplored in visible light photochemistry. A notable exception involves their photoinduced transformation in the presence of a suitable photoexcitable system into the corresponding nitrosoarenes, which subsequently undergo well‐established [4+2] Diels‐Alder cycloadditions to afford 1,2‐*N,O‐*heterocyclic motifs. Building upon these mechanistic insights, a broader and largely unexplored perspective can be envisaged: *N*‐arylhydroxylamines may serve as novel precursors for visible‐light‐induced photoredox transformations beyond nitroso formation. Through rational functionalization or precise modulation of their electronic environments, these compounds may undergo selective N‐O bond activation pathways, leading to the generation of nitrogen‐centered radicals capable of participating in C‐N bond‐forming coupling reactions. This strategy could establish a new paradigm of reactivity for *N*‐arylhydroxylamines, integrating fundamental concepts of hydroxylamine photochemistry with emerging methodologies for the synthesis of structurally diverse *N*‐heterocyclic scaffolds.

## Conflicts of Interest

The authors declare no conflicts of interest.
